# Small extracellular vesicles obtained from hypoxic mesenchymal stromal cells have unique characteristics that promote cerebral angiogenesis, brain remodeling and neurological recovery after focal cerebral ischemia in mice

**DOI:** 10.1007/s00395-021-00881-9

**Published:** 2021-06-08

**Authors:** Jonas Gregorius, Chen Wang, Oumaima Stambouli, Tanja Hussner, Yachao Qi, Tobias Tertel, Verena Börger, Ayan Mohamud Yusuf, Nina Hagemann, Dongpei Yin, Robin Dittrich, Yanis Mouloud, Fabian D. Mairinger, Fouzi El Magraoui, Aurel Popa-Wagner, Christoph Kleinschnitz, Thorsten R. Doeppner, Matthias Gunzer, Helmut E. Meyer, Bernd Giebel, Dirk M. Hermann

**Affiliations:** 1 Department of Neurology and Center for Translational Neuro- and Behavioral Sciences (C-TNBS), University Hospital Essen, University of Duisburg-Essen, Hufelandstraße 55, 45122 Essen, Germany; 2 Institute of Transfusion Medicine, University Hospital Essen, University of Duisburg-Essen, Virchowstraße 179, 45147 Essen, Germany; 3 Institute of Pathology, University Hospital Essen, University of Duisburg-Essen, Essen, Germany; 4 Leibniz Institute for Analytical Sciences (ISAS), Dortmund, Germany; 5 Center of Experimental and Clinical Medicine, University of Medicine and Pharmacy, Craiova, Romania; 6 Department of Neurology, University Medicine Göttingen, Göttingen, Germany; 7 Institute for Experimental Immunology and Imaging, University Hospital Essen, University of Duisburg-Essen, Essen, Germany; 8 Medical Proteom-Center Ruhr University, Bochum, Germany

**Keywords:** Endothelial migration, Microvascular network characteristics, Microvascular remodeling, Neuronal survival, Polymorphonuclear neutrophil, Tube formation

## Abstract

Obtained from the right cell-type, mesenchymal stromal cell (MSC)-derived small extracellular vesicles (sEVs) promote stroke recovery. Within this process, microvascular remodeling plays a central role. Herein, we evaluated the effects of MSC-sEVs on the proliferation, migration, and tube formation of human cerebral microvascular endothelial cells (hCMEC/D3) in vitro and on post-ischemic angiogenesis, brain remodeling and neurological recovery after middle cerebral artery occlusion (MCAO) in mice. In vitro, sEVs obtained from hypoxic (1% O_2_), but not ‘normoxic’ (21% O_2_) MSCs dose-dependently promoted endothelial proliferation, migration, and tube formation and increased post-ischemic endothelial survival. sEVs from hypoxic MSCs regulated a distinct set of miRNAs in hCMEC/D3 cells previously linked to angiogenesis, three being upregulated (miR-126-3p, miR-140-5p, let-7c-5p) and three downregulated (miR-186-5p, miR-370-3p, miR-409-3p). LC/MS–MS revealed 52 proteins differentially abundant in sEVs from hypoxic and ‘normoxic’ MSCs. 19 proteins were enriched (among them proteins involved in extracellular matrix–receptor interaction, focal adhesion, leukocyte transendothelial migration, protein digestion, and absorption), and 33 proteins reduced (among them proteins associated with metabolic pathways, extracellular matrix–receptor interaction, focal adhesion, and actin cytoskeleton) in hypoxic MSC-sEVs. Post-MCAO, sEVs from hypoxic MSCs increased microvascular length and branching point density in previously ischemic tissue assessed by 3D light sheet microscopy over up to 56 days, reduced delayed neuronal degeneration and brain atrophy, and enhanced neurological recovery. sEV-induced angiogenesis in vivo depended on the presence of polymorphonuclear neutrophils. In neutrophil-depleted mice, MSC-sEVs did not influence microvascular remodeling. sEVs from hypoxic MSCs have distinct angiogenic properties. Hypoxic preconditioning enhances the restorative effects of MSC-sEVs.

**Supplementary Information:**

The online version contains supplementary material available at 10.1007/s00395-021-00881-9.

## Introduction

Small extracellular vesicles (sEVs), such as exosomes (50–150 nm), play important roles in intercellular communication [37]. In response to injury, sEVs can promote restorative processes [39]. sEVs prepared from mesenchymal stromal cell (MSC) supernatants have been shown to promote neurological recovery and brain remodeling after focal cerebral ischemia in rats and mice [7, 30, 35, 36]. sEVs possess important characteristics which make them attractive as therapeutics. In contrast to cell therapies, sEVs are not self-replicating and they lack endogenous tumor formation potentials [19]. sEVs can hardly sense environmental conditions, and thus, their biological activity can be predicted more reliably than that of cells [19]. Due to their small size, sEV products can be sterilized by filtration. Hence, their handling is much easier than that of cells. Due to these promising features, sEVs are rapidly approaching clinical trials in human patients [19].

In a head-to-head study in mice, we have previously demonstrated that sEV preparations obtained from supernatants of MSCs cultured under regular, that is, ‘normoxic’, conditions (21% O_2_) equally effectively reduced motor-coordination deficits and increased long-term neuronal survival as their parental MSCs, when they were intravenously administered 24 h after intraluminal middle cerebral artery occlusion (MCAO) [7]. sEV-induced neuroprotection went along with sustained neurogenesis [7]. Neurogenesis and angiogenesis are tightly linked in the ischemic brain [14] and both processes closely accompany successful brain remodeling [12]. Since the cerebral microvasculature, namely endothelial cells, are exposed to intravenously delivered sEVs as first-line targets, the induction of angiogenesis may be instrumental for the capacity of MSC-sEVs to protect ischemic brain tissue. Indeed, evidence of endothelial proliferation was previously found in the ischemic brain after MSC-sEV delivery in mice [7].

The effects of MSC-sEVs on microvessels strongly depend on tissues and pathophysiological states. While MSC-sEV preparations may promote or inhibit angiogenesis in cancer tissues depending on the precise MSC source and tumor microenvironment [6, 18, 41], MSC-sEVs were reported to increase the proliferation and tube formation of cultured human umbilical vein endothelial cells (HUVECs) in an hypoxia-inducible factor-1α (HIF-1α) dependent way [9]. The effects of MSC-sEVs on cerebral microvascular angiogenesis were so far not systematically examined. To evaluate the effects of MSC-sEVs on cerebral angiogenesis, we herein exposed human microvascular endothelial cells (hCMEC/D3) to sEV preparations obtained from MSCs of two randomly selected healthy human donors, which had been cultured under regular ‘normoxic’ conditions (21% O_2_) or hypoxic conditions (1% O_2_), or to sEVs obtained from MSC culture media, which contain platelet lysates. In additional studies, mice that underwent intraluminal MCAO likewise received sEV preparations from ‘normoxic’ MSCs, from hypoxic MSCs or from cell culture media. In some subgroups, polymorphonuclear neutrophil leukocytes (PMN), which have previously been shown to mediate acute neuroprotective effects of MSC-sEVs [30], were depleted. We report that sEVs obtained from hypoxic, but not ‘normoxic’ MSCs or culture media induce angiogenesis as indicated by endothelial proliferation, transwell migration or tube formation assays in vitro and microvascular network characteristic analysis in vivo. Interestingly, sEVs from hypoxic MSCs regulated a distinct set of hitherto unrecognized miRNAs in hCMEC/D3 cells that have been linked to angiogenesis. Liquid chromatography/tandem mass spectrometry (LC/MS–MS) revealed previously unnoted proteins in sEVs from hypoxic MSCs that mediate their angiogenic properties. In vivo, the proangiogenic effects of hypoxic MSC-sEVs were abolished by PMN depletion, indicating that PMNs support the restorative effects of MSC-sEVs in the post-acute stroke phase.

## Materials and methods

### Propagation, isolation and characterization of MSCs and MSC-sEVs

Following previously described protocols [17, 25], we raised MSCs from bone marrow samples of seven randomly selected healthy human donors (MSC sources 41.5, 16.3, 117.3, 126.7, 142.1, 152.6, 153.3), some of which (sources 41.5, 16.3) have previously been characterized by us in an in vivo intraluminal MCAO model in the acute stroke phase [30]. These cells exhibit *bona fide* MSC characteristics, expressing cell surface antigens CD44, CD73, CD90 and CD105 and lacking endothelial and hematopoietic marker proteins (that is, CD31, CD34 and CD45) (Suppl. Fig. 1). The cells have proven differentiation capacity in osteogenic and adipogenic lineages [30]. sEVs were obtained from supernatants, while MSCs were cultured under regular ‘normoxic’ (21% O_2_; sEV_normoxic_) or hypoxic (1% O_2_; sEV_hypoxic_) conditions over 48 h. Hypoxic preconditioning was used to mimic the effects of stroke-associated hypoxia on the restorative capacity of MSCs. Hypoxia did not result in any histological signs of structural MSC cell injury. Hypoxia did not influence MSC viability assessed by 3-(4,5-dimethylthiazol-2-yl)-2,5-diphenyltetrazolium bromide (MTT). As MSC culture medium, Dulbecco’s Modified Eagle’s Medium was used containing low glucose (DMEM low glucose, Lonza, Basel, Switzerland) supplemented with 10% platelet lysate obtained from pooled healthy donors (Institute of Transfusion Medicine, University Hospital Essen), 100 U/ml penicillin/streptomycin/glutamine (Gibco/Life Technologies, Carlsbad, CA, USA) and 5 IU/ml heparin (ratiopharm, Ulm, Germany). sEVs were isolated utilizing an optimized polyethylene glycol 6000 precipitation protocol followed by ultracentrifugation as described before [3, 21]. Three independent preparations of 41.5 MSC-sEVs were performed under ‘normoxic’ (21% O_2_; sEV_normoxic_) and hypoxic (1% O_2_; sEV_hypoxic_) conditions, which were each evaluated in proliferation, transwell migration, and tube formation assays. All MSC-sEV preparations were characterized according to the current minimal recommendations of the International Society of Extracellular Vesicles (ISEV) [25]. Particle concentration and size of sEV preparations were analyzed using nanoparticle tracking analysis (Suppl. Table 1). Protein contents of sEVs were determined using the bicinchoninic acid (BCA) assay (Pierce, Rockford, IL, USA) (Suppl. Table 1). Using Western blots, we had previously shown the presence of the exosome markers CD9, CD63, CD81, and syntenin and the absence of the cytosolic markers calnexin and prohibitin in our sEV preparations [30]. By imaging flow cytometry (IFC) using the AMNIS ImageStreamX Mark II Flow Cytometer (Luminex, Seattle, WA, USA), we now showed that the number of CD9^+^, CD63^+^ and CD81^+^ sEVs varied in different sEV preparations from the same source, independent of whether sEVs were collected under ‘normoxic’ or hypoxic conditions (Suppl. Fig. 2). For staining, anti-CD9 (EXBIO, Vestec, Czech Republic), anti-CD63 (EXBIO), and anti-CD81 (Beckman Coulter, Brea, CA, USA) or corresponding isotype antibodies were used [10, 28]. Transmission electron microscopy confirmed the presence of CD9^+^ sEVs that had the appearance (double membrane-like configuration) and size (50–160 nm diameter) of exosomes (Suppl. Fig. 3). Data were analyzed as described previously using IDEAS software (version 6.2).

### Endothelial cell culture

hCMEC/D3 cells (kindly provided by Dr. Pierre-Olivier Couraud, Paris) from passage 28 to 34 were cultivated in Endothelial Cell Growth Basal Medium-2 (EBM-2; Lonza) containing 5% fetal bovine serum (FBS) (Gibco/Life Technologies), 1% Hepes buffer (10 mM, Gibco/Life Technologies), 1% chemically defined lipid concentrate (Gibco/Life Technologies), 1% penicillin/streptomycin solution (Gibco/Life Technologies), 0.5% ascorbic acid (5 µg/mL), 0.5% human basic fibroblast growth factor (bFGF; 1 ng/mL; Sigma-Aldrich, St. Louis, MO, USA), and 0.05% hydrocortisone (1.4 µM, Sigma-Aldrich) in a humidified atmosphere at 37 °C containing 21% O_2_ and 5% CO_2_.

### Proliferation assay

Samples of 3 × 10^5^ hCMEC/D3 cells were seeded in 6-well plates containing EBM-2 medium complemented with 1.25% FBS (other supplements as above). sEVs at various concentrations (0–250 µg/mL) obtained from (a) MSC culture medium (DMEM low, Lonza) supplemented with 10% platelet lysate (sEV_platelet_), (b) supernatants of MSCs cultured under regular ‘normoxic’ conditions (21% O_2_; sEV_normoxic_), or (c) supernatants of MSCs cultured under hypoxic conditions (1% O_2_; sEV_hypoxic_) were added. Forty-eight hours later, cells were stained with 0.4% tryptan blue (Sigma-Aldrich). The number of viable cells was determined using an automatic cell counter (EVE Automatic Cell Counter; NanoEnTek, Waltham, MA, USA).

### Transwell migration assay

Similarly, 3 × 10^4^ hCMEC/D3 cells were seeded into the upper compartment of polycarbonate membrane transwell inserts (8.0 µm pores; GE Healthcare Life Sciences, Chicago, IL, USA) in 24-well plates containing EBM-2 medium complemented with 1.25% FBS (other supplements as above). (a) sEV_platelet_, (b) sEV_normoxic_ or (c) sEV_hypoxic_ (as above) at various concentrations (0–250 µg/mL) were added into the lower compartment. Twenty-four hours later, remaining cells on top of the filter were removed with 0.1 M phosphate-buffered saline (PBS; Gibco/Life Technologies) soaked cotton swabs and cells that crossed the filter were fixed with 4% PFA followed by Hoechst staining. For image acquisition, filters were cut and mounted on glass slides using Fluoromount medium (Thermo Fisher Scientific, Waltham, MA, USA). Images were taken at 20X magnification using a fluorescence microscope (BX51, Olympus, Shinjuku, Japan). The number of migrated cells was counted using ImageJ software (National Institutes of Health, Bethesda, MD, USA) in a total of 7 regions of interest (ROI) per filter measuring 600 × 400 µm.

### Tube formation assay

Likewise, 3 × 10^4^ hCMEC/D3 cells were seeded in 96-well plates precoated with Matrigel (Corning, Corning, NY, USA). (a) sEV_platelet_, (b) sEV_normoxic_ or (c) sEV_hypoxic_ (as above) at various concentrations (0–250 µg/mL) were added. Twenty hours later, images were taken using a digital inverted microscope with 4X magnification (AMG EVOS fl; Advanced Microscopy Group, Bothell, WA, USA). The number of closed tubes, total tube length, branching point number, and the mean length of branches between two branching points were evaluated using ImageJ software in a ROI measuring 4,300 × 3,225 µm.

### MTT assay of cell viability

Similarly, 3 × 10^4^ hCMEC/D3 cells seeded in sextuplicates in 96-well plates were incubated for 24 h. After washing twice in 0.1 M PBS, cells were cultured under regular conditions (21% O_2_) or oxygen–glucose deprivation (OGD) that was induced by transferring the cells in glucose-free DMEM medium (Gibco/Life Technologies) and incubated at 1% O_2_ in a hypoxia chamber (Toepffer Lab Systems, Göppingen, Germany). (a) sEV_platelet_, (b) sEV_normoxic_ or (c) sEV_hypoxic_ (as above) at various concentrations (0–250 µg/mL) were added. After 24 h, hCMEC/D3 cells exposed to OGD were washed and cell medium was replaced with regular medium, to which sEVs were added as before. Viable cells were labeled with 10% MTT. Cells were fixed with DMSO (Sigma-Aldrich). Light absorbance was measured using a microplate absorbance reader (iMark™; Bio-Rad Laboratories, Hercules, CA, USA) at 570 nm wavelength.

### Intraluminal MCAO and MSC-sEV delivery

Animal experiments were performed with local government approval (Northrhine-Westphalian State Agency for Nature, Environment and Consumer Protection, Recklinghausen; permission G1680/18) in accordance to E.U. guidelines (Directive 2010/63/EU) for the care and use of laboratory animals and reported based on Animal Research: Reporting of In Vivo Experiments (ARRIVE) guidelines. Experiments were strictly randomized. The experimenter performing the animal experiments and histochemical studies (C.W.) was fully blinded at all stages of the study by another researcher (N.H.) preparing the vehicle and sEV solutions. These solutions received dummy names (solution A, B, C, and D, or A, B, C, D, and E), which were unblinded after termination of the study. Animals were kept in a regular inverse 12 h:12 h light/dark cycle in groups of 5 animals/cage.

Focal cerebral ischemia was induced in male C57BL6/j mice (8–10 weeks; 22–25 g; Harlan Laboratories, Darmstadt, Germany) anesthetized with 1.0–1.5% isoflurane (30% O_2_, remainder N_2_O) by 40 min left-sided intraluminal MCAO [30]. Rectal temperature was maintained between 36.5 and 37.0 °C using a feedback-controlled heating system (Fluovac; Harvard apparatus, Holliston, MA, USA). Cerebral blood flow was recorded by laser Doppler flow (LDF) measurement using a flexible probe attached to the skull overlying the middle cerebral artery territory core. The left common and external carotid arteries were isolated and ligated, and the internal carotid artery was temporarily clipped. A silicon-coated nylon monofilament (Doccol Corp., Sharon, MA, USA) was introduced through a small incision into the common carotid artery and advanced to the carotid bifurcation for MCAO. Reperfusion was initiated by monofilament removal. Following termination of the surgeries, wounds were carefully sutured. For analgesia, buprenorphine (0.1 mg/kg; Reckitt Benckiser, Slough, UK) was s.c. administered 30 min before MCAO. For anti-inflammation, animals received daily i.p. carprofen (4 mg/kg; Bayer Vital, Leverkusen, Germany) injections post-MCAO.

Twenty-four, 72 and 120 h post-MCAO, 200 µl of (a) vehicle (normal saline), (b) sEV_platelet_, (c) sEV_normoxic_ or (d) sEV_hypoxic_ (equivalent released by 2 × 10^6^ cells, in normal saline) were administered through the animals’ tail vein, as reported previously [7, 30]. In animals sacrificed 14 days post-MCAO, 100 µg control isotype IgG (clone 2A3; BioXCell, West Lebanon, NH, USA) or 100 µg anti-Ly6G antibody (clone 1A8; BioXCell) were intraperitoneally administered at 24 h, 72 h, 120 h, and 168 h post-MCAO for PMN depletion, in parallel with the delivery of 100 μg anti-rat kappa immunoglobulin light chain (clone MAR 18.5; BioXCell) at 48, 96, and 144 h, which was administered for the augmentation of PMN sequestering. Neurological deficits were evaluated at 1, 7, 14, 21, 28, 35 and 42 days post-MCAO by Clark scores.

Mice were excluded from the study when they met one of the following exclusion criteria: (1) prolonged surgery duration > 20 min, (2) drop of Laser Doppler flow < 75% after monofilament insertion, (3) lack of reperfusion after monofilament withdrawal or (4) > 20% weight loss, respiratory abnormalities (central apneas) or death. In mice sacrificed at 14 days post-MCAO, a total of 6 mice (1 isotype IgG/vehicle, 2 isotype IgG/sEV_normoxic_, 1 isotype IgG/sEV_hypoxic_, 1 anti-Ly6G/vehicle, 1 anti-Ly6G/sEV_hypoxic_), and in mice sacrificed at 56 days post-MCAO, at total of 5 mice (1 vehicle, 2 sEV_platelet_, 1 sEV_normoxic_, 1 sEV_hypoxic_) were excluded. Animals excluded were substituted by new animals. For animals sacrificed at 56 days, 10 mice receiving vehicle, 6 mice receiving sEV_platelet_, 9 mice receiving sEV_normoxic_, and 9 mice receiving sEV_hypoxic_, and for animals sacrificed at 14 days, 6 mice receiving isotype IgG/vehicle, 5 mice receiving isotype IgG/sEV_normoxic_, 8 mice receiving isotype IgG/sEV_hypoxic_, 7 mice receiving anti-Ly6G/vehicle, and 7 mice receiving anti-Ly6G/sEV_hypoxic_ were prepared.

Fourteen or 56 days post-MCAO, mice were deeply anesthetized and sacrificed by transcardiac perfusion with 0.1 M phosphate-buffered saline (PBS) and 4% paraformaldehyde in 0.1 M PBS. For imaging of the reperfusion status of the brain tissue at the onset of MSC-sEV treatment, an additional control group of 5 mice exposed to intraluminal MCAO that were sacrificed after 24 h was prepared. These mice did not obtain any sEV or antibody treatment.

### FITC-albumin hydrogel perfusion and whole-brain clearing for 3D light sheet fluorescence microscopy (LSFM)

In animals sacrificed at 14 days post-MCAO, 10 ml of a hand-warm (30 °C) 2% gelatin hydrogel containing 0.1% FITC-conjugated albumin, which had been filtered using Whatman filter paper (GE Healthcare Life Science, Little Charfont, U.K.) and was protected from light, was transcardially infused into the animals’ aorta immediately following PFA infusion. Brains were subsequently removed, post-fixed overnight at 4 °C in 4% PFA in 0.1 M PBS and dehydrated through a 30%, 60%, 80%, and 100% tetrahydrofuran (THF; Sigma-Aldrich) gradient [22]. Brain clearing was achieved with ethyl cinnamate (ECI; Sigma-Aldrich).

### 3D LSFM and microvasculature analysis

The FITC-albumin labeled vasculature of cleared brains was scanned by a light sheet microscope (Ultramicroscope Blaze, LaVision BioTec, Göttingen, Germany) that was equipped with a 488 nm laser. Horizontal overview images of the cleared brain were taken using a 1.6 × objective. Serial images of the striatum and cortex were acquired at 2 µm steps using a 6.4 × objective. In each animal, two regions of interest (ROI) measuring 500 µm × 500 µm × 1000 µm (in the *X*, *Y* and *Z* planes, respectively) in the dorsolateral striatum were analyzed using Imaris (Bitplane, Zurich, Switzerland) software with 3D rendering software package, as described previously [22]. Following image segmentation, skeletonization and 3D reconstruction, a comprehensive set of microvascular network characteristics, that is, microvascular length density, branching point density, mean branch length between two branching points, and microvascular tortuosity, were determined.

### Brain volumetry

Twenty-μm-thick coronal brain cryostat sections of mice sacrificed at 56 days post-MCAO collected at 1 mm intervals across the forebrain were stained with cresyl violet. Using ImageJ software, the striatum volume and brain volume ipsilateral and contralateral to the stroke were determined, of which ipsilateral-to-contralateral volume ratios were formed.

### Immunohistochemical analysis of microvascular density, neuronal survival, and astrocytic scar formation

Twenty-µm-thick sections obtained from the rostrocaudal level of the bregma of the same mice were rinsed three times for 5 min in 0.1 M PBS and immersed in 0.1 M PBS containing 0.1% triton (PBS-T) containing 10% normal donkey serum for 10 min. Sections were incubated overnight at 4 °C in monoclonal rat anti-CD31 (#550,274; clone MEC13.3; BD Biosciences, Heidelberg, Germany), monoclonal rabbit anti-neuronal nuclear antigen (NeuN; #ab177487; clone EPR12763; Abcam), or monoclonal rat anti-glial fibrillary acidic protein (GFAP; #13–0300; clone 2.2B10; Thermo Fisher Scientific) antibody. After rinsing, sections were incubated for 1 h at room temperature in an appropriate secondary antibody solution. Nuclei were counterstained with Hoechst 33,342 (#B2261; Sigma-Aldrich). Immunofluorescence was evaluated using a Zeiss AxioOberver.Z1 inverted microscope (Carl Zeiss, Jena, Germany) by measuring the length of CD31^+^ microvascular profiles in three regions of interest (ROI; each measuring 195 μm × 195 μm) each in the most lateral part of the ischemic striatum directly adjacent to the external capsule, which represents the core of the middle cerebral artery territory, in the peri-infarct parietal cortex and the infarct-remote motor cortex. Mean values were calculated for these ROI. In adjacent sections at the bregma level, the total number of NeuN^+^ neurons was counted in the ischemic and contralateral striatum. By dividing values determined in the ischemic and contralateral striatum, the ratio of surviving neurons was calculated for each animal. GFAP staining was evaluated by analyzing the area of GFAP^+^ astrocytic scar on coronal sections for each animal.

### NanoString miRNA expression analysis

hCMEC/D3 cells seeded in 6-well plates were exposed to (a) control conditions, (b) sEV_platelet_, (c) sEV_normoxic_ or (d) sEV_hypoxic_ (50 µg/mL each) for 24 h, followed by RNA extraction using the miRNeasy FFPE kit (Qiagen, Hilden, Germany). RNA concentration was measured using a Qubit 2.0 fluorometer (Life Technologies). RNA integrity was assessed using a Fragment Analyzer (Advanced Analytical Inc., Ames, IA, USA) using the DNF-489 standard sensitivity RNA analysis kit.

The commercially available nCounter miRNA Expression Assay v2.1 (NanoString Technologies, Seattle, WA, USA) containing probes and miRTags for 800 important miRNAs described in the context of cell proliferation and cancer was chosen for miRNA expression analysis. Five potential reference genes (ACTB, B2M, GAPDH, RPL19, RPLP0) were included in the CodeSet for biological normalization purposes. Probe sets and miRTags for each target in the CodeSet were designed and synthesized at NanoString Technologies. Samples containing 100 ng total RNA were analyzed for each sample (in a final volume of 3 µl). The sample preparation in the nCounter Prep Station (NanoString Technologies) was carried out using the high-sensitivity protocol (3 h preparation). The cartridges were measured in 555 fields of view in the nCounter Digital Analyzer (NanoString Technologies).

Data processing was performed using the R statistical programming environment (v3.2.3) using the NanoStringNorm and the *NAPPA* package (NanoString Technologies), respectively. Considering the counts obtained for positive control probe sets, raw NanoString counts for each gene were subjected to a technical factorial normalization, carried out by subtracting the mean counts plus two standard deviations from the CodeSet inherent negative controls. Afterwards, a biological normalization using the top 100 expressed miRNAs was performed. In brief, gene expression stability measures (M) were calculated using the geNorm algorithm. All sample counts were normalized against the geometric mean of the normalization counts. To overcome basal noise, counts with *p* > 0.05 versus negative controls in one-sided Wilks *t* tests plus two standard deviations were interpreted as not expressed. Normal distribution of data was evaluated using Shapiro–Wilk tests. Based on the results, either parametric or non-parametric were applied, as appropriate, and p values were adjusted for the false discovery rate.

### Liquid chromatography/tandem mass spectrometry (LC/MS–MS)

Proteins of (a) sEV_platelet_, (b) sEV_normoxic_ or (c) sEV_hypoxic_ (50 µg/mL each) were isolated using C02-micro-80 S-Trap filters (Protifi, Huntington, NY, USA). To this end, 25 μg protein of each sEV lysate was solubilized in 5% sodium dodecyl sulfate (SDS) triethylammonium bicarbonate (pH 7.55). Disulfide bonds were broken using 20 mM DTT at 60 °C for 30 min. After cooling to room temperature, the sample was alkylated in the dark with 20 mM iodoacetamide for 30 min. Afterwards, phosphoric acid was added to a final concentration of 1.2%. Following resuspension, six volume equivalents of S-TRAP binding buffer (90% methanol; 100 mM triethylammonium bicarbonate, pH 7.19) were added. The sample was loaded on an S-Trap filter, centrifuged for 30 s at 4,000 rpm, and the flow-through discarded. The filter bound protein was washed 3 times according to the previous step; the filter was transferred into a fresh tube and incubated with the digest solution (50 mM ammonium bicarbonate, pH 7.8, 0.2 M guanidine hydrochloride and trypsin; Sigma-Aldrich) in a 20:1 ratio at 47 °C for 2 h. The elution of peptides was ensured by three elution steps for 1 min and 4,000 rpm (step 1: 50 mM ammonium bicarbonate, step 2: 0.2% formic acid in H_2_O, and step 3: 0.2% formic acid and 50% acetonitrile [ACN]). The obtained solution was frozen at − 80 °C, and the buffer exchanged against 1% trifluoroacetic acid (TFA). Post-digestion quality control was performed on a monolithic high-performance liquid chromatography (HPLC) as described previously [4].

To achieve a higher identification rate, samples were dried under vacuum, resuspended in 10 mM ammonium acetate (pH 8.0; buffer A), and fractionated on an Ultimate 3000 LC (Thermo Fisher Scientific). Peptides were separated on a 1 mm × 150 mm C18 (ZORBAX 300SB-C18, pore size 300 Å, 5 μm particle size; Agilent Technologies, Santa Clara, CA, USA) column with a 45 min LC gradient ranging from 3 to 45% buffer B (84% ACN in 10 mM ammonium acetate, pH 8.0) at a flow rate of 12.5 μL/min resulting in 10 fractions of each sample. Each fraction was dried and resuspended in 15 μL of 0.1% TFA for nano-liquid chromatography–tandem mass spectrometry (LC−MS/MS) analysis, which was performed using an Ultimate 3000 nano-LC system coupled to an LTQ Orbitrap Velos instrument (Thermo Fisher Scientific) using pre-column trap columns (100 μm × 2 cm, C18 Acclaim Pepmap viper) and main columns (75 μm × 50 cm, C18 Acclaim Pepmap viper; Thermo Fisher Scientific). A 118 min LC gradient ranging from 3 to 42% of buffer B (84% ACN, 0.1% TFA) at a flow rate of 250 nL/min was used. The ten most intense ions were fragmented and the data qualitatively analyzed. Unfractionated samples were prepared and analyzed the same way on an Orbitrap Fusion Lumos (Thermo Fisher Scientific). MS survey scans were acquired from 300 to 1500 m/z at a resolution of 60,000, using an AGC target value of 4 × 10^5^ and a maximum injection time of 70 ms. Here, a 185 min LC gradient ranging from 3 to 35% of buffer B (84% ACN, 0.1% TFA) at a flow rate of 250 nL/min was used, and the ten most intense ions were fragmented and the data analyzed. Normal distribution of data was evaluated using Shapiro–Wilk tests.

### Statistical analysis

For statistical analysis, a SPSS for Windows software package (version 22.0) was used. Data were evaluated by one-way or two-way analysis of variance (ANOVA) followed by least significant differences (LSD) tests (≥ 3 groups, normally distributed data) or two-sided *t* tests (2 groups, normally distributed data) or by Kruskal–Wallis tests (≥ 3 groups, non-normally distributed data) or Mann–Whitney *U* tests (2 groups, non-normally distributed data), as adequate. Results are shown as mean ± standard deviation (SD) values (angiogenesis assays) or as median values ± interquartile ranges (IQR) with minimum and maximum data as whiskers (in vivo data; miRNA expression analyses). *P* values < 0.05 were considered significant. Data supporting our findings are available from the corresponding author on reasonable request.

## Results

### sEV preparations obtained from hypoxic MSCs dose-dependently increase cerebral microvascular endothelial cell proliferation

**Fig. 1:**
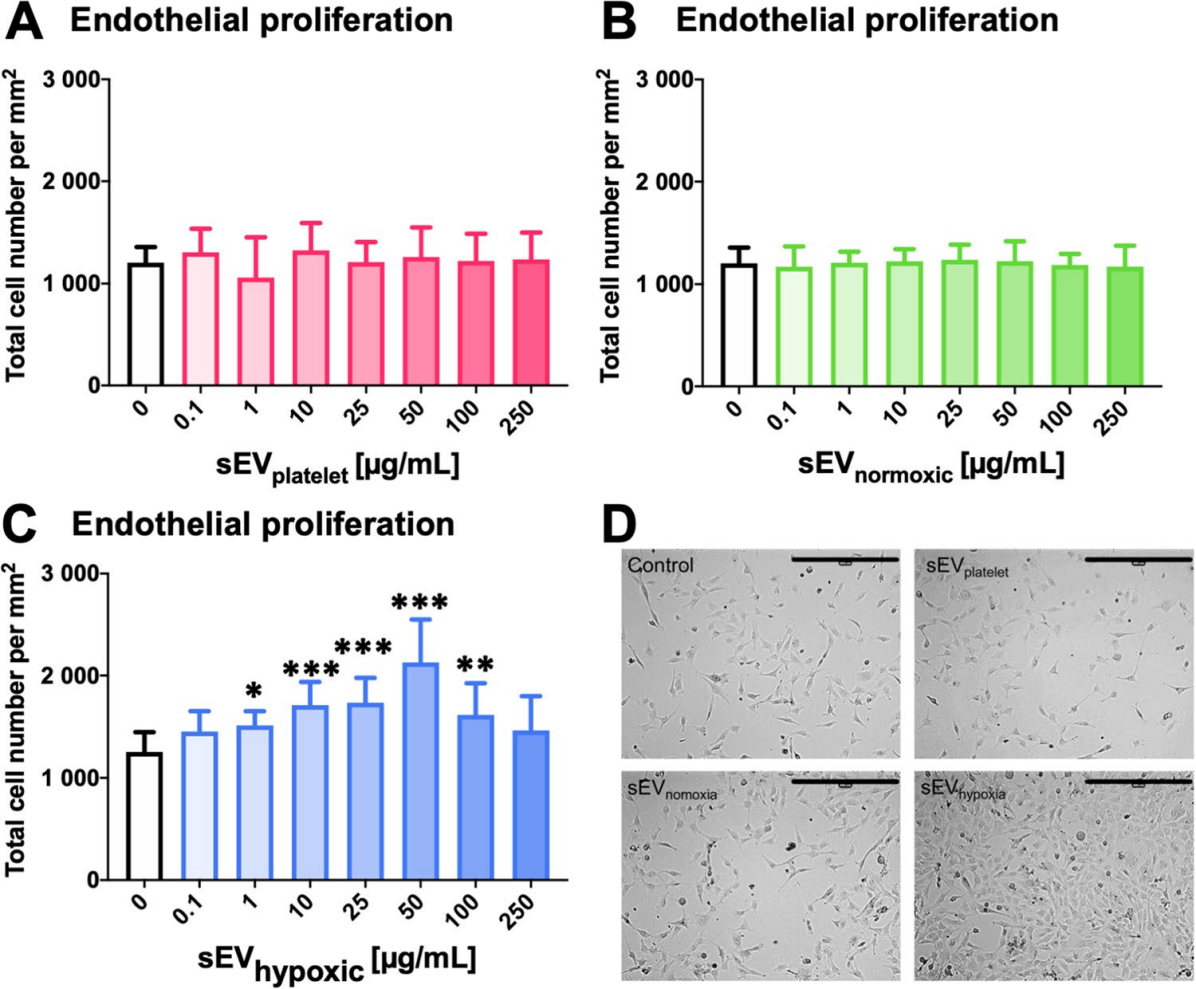
Small extracellular vesicles (sEVs) obtained from mesenchymal stromal cells (MSCs) cultured under hypoxic conditions increase cerebral microvascular endothelial cell proliferation. Relative number of human microvascular endothelial cells (hCMEC/D3) after exposure to different concentrations of **A** sEVs obtained from MSC culture media that contain platelet lysates (sEV_platelet_), **B** sEVs obtained from MSCs ( source 41.5) cultured under regular ‘normoxic’ conditions (21% O_2_; sEV_normoxic_) or **C** sEVs obtained from MSCs (source 41.5) cultured under hypoxic conditions (1% O_2_; sEV_hypoxic_). In **D**, representative microphotographs for hCMEC/D3 cells exposed to control conditions or sEVs at a concentration of 50 µg/mL are shown. Data are mean ± SD values (*n* = 9 independent experiments). *p < 0.05, **p < 0.01, ***p < 0.001 compared with control. Scale bar: 400 µm in **D**

During angiogenesis, endothelial cells proliferate, migrate, and form capillary tubes [14]. To account for these processes, we evaluated these features using the hCMEC/D3 cell line. While sEVs obtained from MSC cell culture media (sEV_platelet_; Fig. 1A, D) or sEVs from ‘normoxic’ MSCs (sEV_normoxic_, source 41.5; Fig. 1B, D) did not influence endothelial proliferation at any dose examined, sEVs obtained from hypoxic MSCs (sEV_hypoxic_, again source 41.5) dose-dependently increased the proliferation of hCMEC/D3 cells (Fig. 1C, D). This increase was statistically significant between doses of 1 and 100 µg/mL and reached maximum levels at 50 µg/mL. Higher doses (250 µg/mL) did not promote endothelial proliferation. A separate analysis of three independent preparations of 41.5 MSC-sEVs revealed that sEV_hypoxic_ consistently increased hCMEC/D3 cell proliferation, whereas sEV_platelet_ or sEV_normoxic_ did not (Suppl. Fig. 4A–C). In view of these findings, we performed additional studies using sEVs obtained from MSCs of another donor (source 16.3). Again sEV_hypoxic_, but not sEV_platelet_ or sEV_normoxic_ increased hCMEC/D3 cell proliferation (Suppl. Fig. 5).

### sEV preparations obtained from hypoxic MSCs dose-dependently increase cerebral microvascular endothelial cell migration and tube formation

**Fig. 2:**
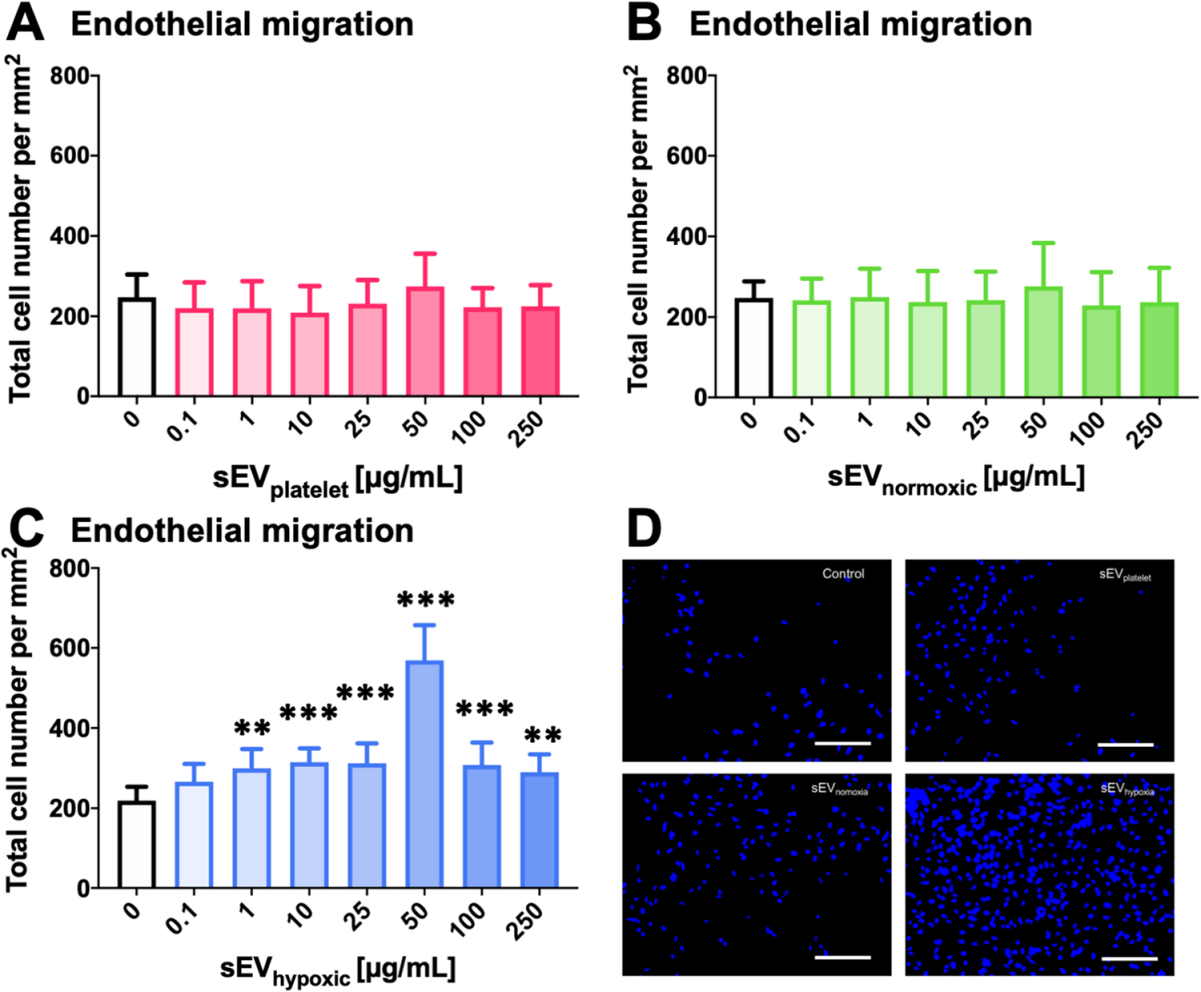
sEVs from hypoxic MSCs increase cerebral microvascular endothelial cell migration. Relative number of migrating hCMEC/D3 cells, determined in a modified Boyden chamber transwell migration assay, after exposure to different concentrations of **A** sEVs obtained from MSC culture media that contain platelet lysates (sEV_platelet_), **B** sEVs obtained from MSCs ( source 41.5) cultured under ‘normoxic’ conditions (21% O_2_; sEV_normoxic_) or **C** sEVs obtained from MSCs (source 41.5) cultured under hypoxic conditions (1% O_2_; sEV_hypoxic_). In **D**, representative microphotographs for hCMEC/D3 cells exposed to control conditions or sEVs at a 50 µg/mL concentration are shown. Data are mean ± SD values (*n* = 9 independent experiments). *p < 0.05, **p < 0.01, ***p < 0.001 compared with control. Scale bars: 125 µm in **D**

**Fig. 3:**
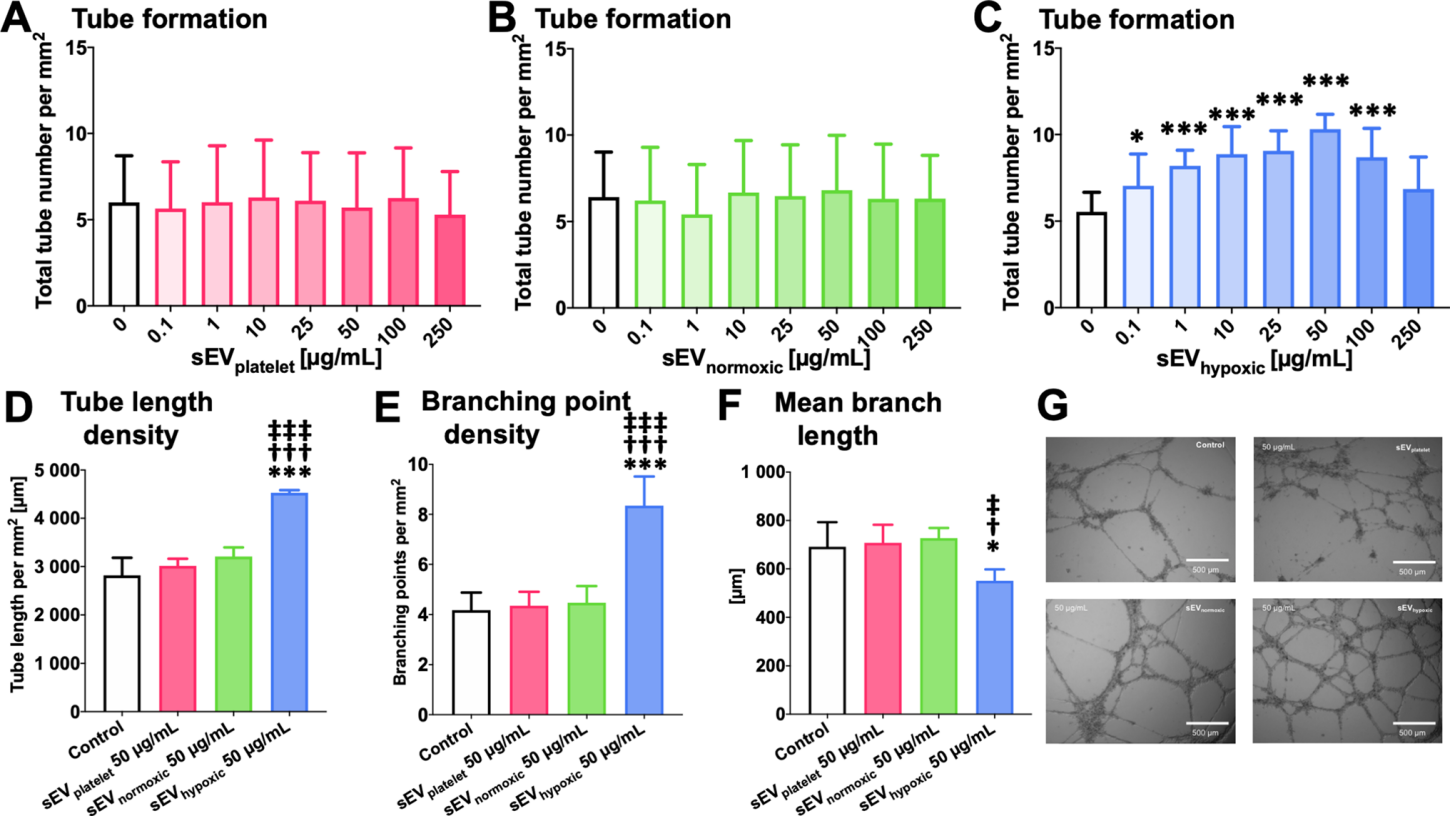
sEVs from hypoxic MSCs increase the tube formation of cerebral microvascular endothelial cells. Relative tube number, evaluated in a Matrigel-based tube formation assay, of hCMEC/D3 cells exposed to different concentrations of **A** sEVs obtained from MSC culture media that contain platelet lysates (sEV_platelet_), **B** sEVs obtained from MSCs ( source 41.5) cultured under regular ‘normoxic’ conditions (21% O_2_; sEV_normoxic_) or **C** sEVs obtained from MSCs (source 41.5) cultured under hypoxic conditions (1% O_2_; sEV_hypoxic_). **D** Microvascular tube length density, **E** microvascular branching point density and **F** mean branch length between two branching points of hCMEC/D3 cells exposed to control conditions or sEV_platelet_, sEV_normoxic_ (MSC source 41.5) or sEV_hypoxic_ (MSC source 41.5) at a concentration of 50 µg/mL (*n* = 3–9 independent experiments). In **G**, representative microphotographs for hCMEC/D3 cells exposed to control conditions or 50 µg/mL sEVs are shown. Data are mean ± SD values (*n* = 9 independent experiments [in **A**–**C**], 3–9 independent experiments [in **D**–**F**]). *p < 0.05, ***p < 0.001 compared with control/^†^*p* < 0.05, ^†††^*p* < 0.001 compared with sEV_platelet_/^‡^*p* < 0.05, ^‡‡‡^*p* < 0.001 compared with sEV_normoxic_. Scale bars: 500 µm

Likewise, sEV_hypoxic_, but not sEV_platelet_ or sEV_normoxic_ obtained from 41.5 MSCs dose-dependently increased hCMEC/D3 cell transwell migration (Fig. 2A–C) and tube formation (Fig. 3A–C). Increases in transwell migration and tube formation were statistically significant at doses of 1–250 µg/mL and 0.1–100 µg/mL, respectively. Maximum effects were noted at doses of 50 µg/mL. In-depth analysis of tube formation using the 50 µg/mL dose revealed that sEV_hypoxic_ obtained from 41.5 MSCs increased the microvascular tube length density (Fig. 3D), increased the branching point density (Fig. 3E) and reduced the mean branch length between two branching points (Fig. 3F), when compared with control conditions, sEV_platelet_ or sEV_normoxic_. Representative transwell migration assays are shown in Fig. 2D, and representative tube formation assays in Fig. 3G. A separate analysis of the three preparations of 41.5 MSC-sEVs revealed that sEV_hypoxic_ consistently increased hCMEC/D3 cell migration and tube formation, whereas sEV_platelet_ or sEV_normoxic_ did not (Suppl. Figs. 6A–C, 7A–C). In view of these findings, we also performed studies using sEVs obtained from MSCs of six other donors (sources 16.3, 117.3, 126.7, 142.1, 152.6, and 153.3). Again, sEV_hypoxic_, but not sEV_platelet_ or sEV_normoxic_ consistently increased hCMEC/D3 cell migration and tube formation (Suppl. Fig. 8A–E).

### sEV preparations obtained from hypoxic MSCs promote the post-ischemic survival of cerebral microvascular endothelial cells

**Fig. 4:**
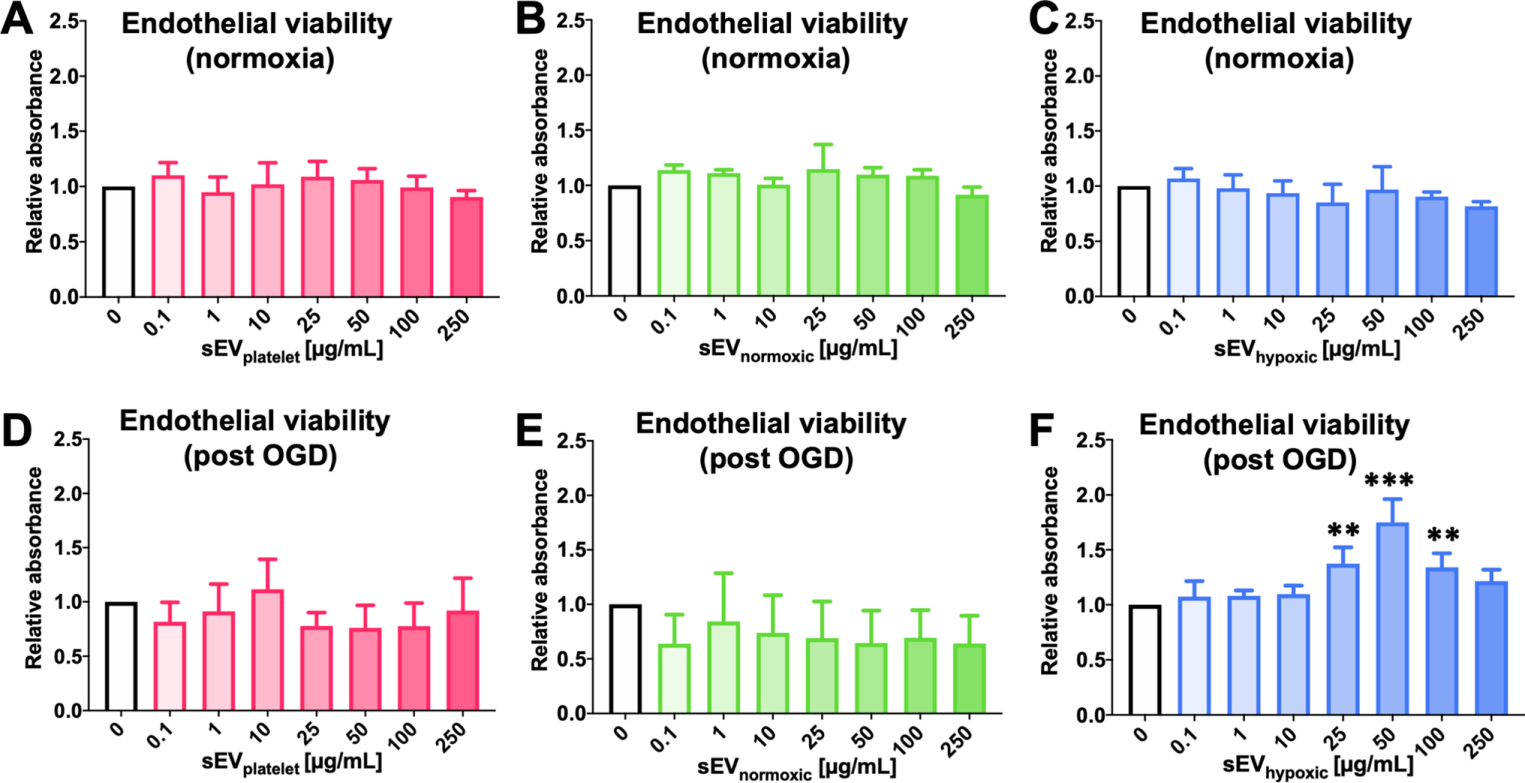
sEVs from hypoxic MSCs increase the survival of cerebral microvascular endothelial cells exposed to oxygen–glucose deprivation (OGD), but do not influence the viability of cells cultured under regular ‘normoxic’ conditions. Relative absorbance of hCMEC/D3 cells cultured under (**A–C**) regular ‘normoxic’ conditions (21% O_2_) or (**D–F**) 24 h OGD (1% O_2_, glucose deprivation) followed by 6 h reoxygenation (21% O_2_)/glucose recultivation, determined in a 3-(4,5-dimethylthiazol-2-yl)-2,5-diphenyltetrazolium bromide (MTT) assay after exposure to different concentrations of **A**, **D** sEVs obtained from MSC culture media that contain platelet lysates (sEV_platelet_), **B**, **E** sEVs obtained from MSCs ( source 41.5) cultured under regular ‘normoxic’ conditions (21% O_2_; sEV_normoxic_) or **C**, **F** sEVs obtained from MSCs (source 41.5) cultured under hypoxic conditions (1% O_2_; sEV_hypoxic_). Data are mean ± SD values (*n* = 3 independent experiments [in **A**–**E**], 8 independent experiments [in **F**]). **p < 0.01, ***p < 0.001 compared with control

In ischemic tissues, the balance of endothelial proliferation and degeneration crucially determines vascular remodeling [14]. Hence, we evaluated the effects of MSC-sEVs on the viability of hCMEC/D3 cells cultured under regular ‘normoxic’ conditions and hCMEC/D3 cells exposed to 24 h OGD followed by 6 h reoxygenation/recultivation by MTT assays. The viability of hCMEC/D3 cells cultured under regular ‘normoxic’ conditions (21% O_2_) was not influenced by sEVs at any of the doses examined (Figs. 4A–C; Suppl. Fig. 8F), indicating lack of sEV toxicity. On the other hand, sEV_hypoxic_, but not sEV_platelet_ or sEV_normoxic_ dose-dependently increased the survival of hCMEC/D3 cells exposed to 24 h OGD followed by 6 h reoxygenation/recultivation (Fig. 4D–F).

### sEV preparations obtained from hypoxic MSCs regulate a distinct set of miRNAs in cerebral microvascular endothelial cells

**Fig. 5:**
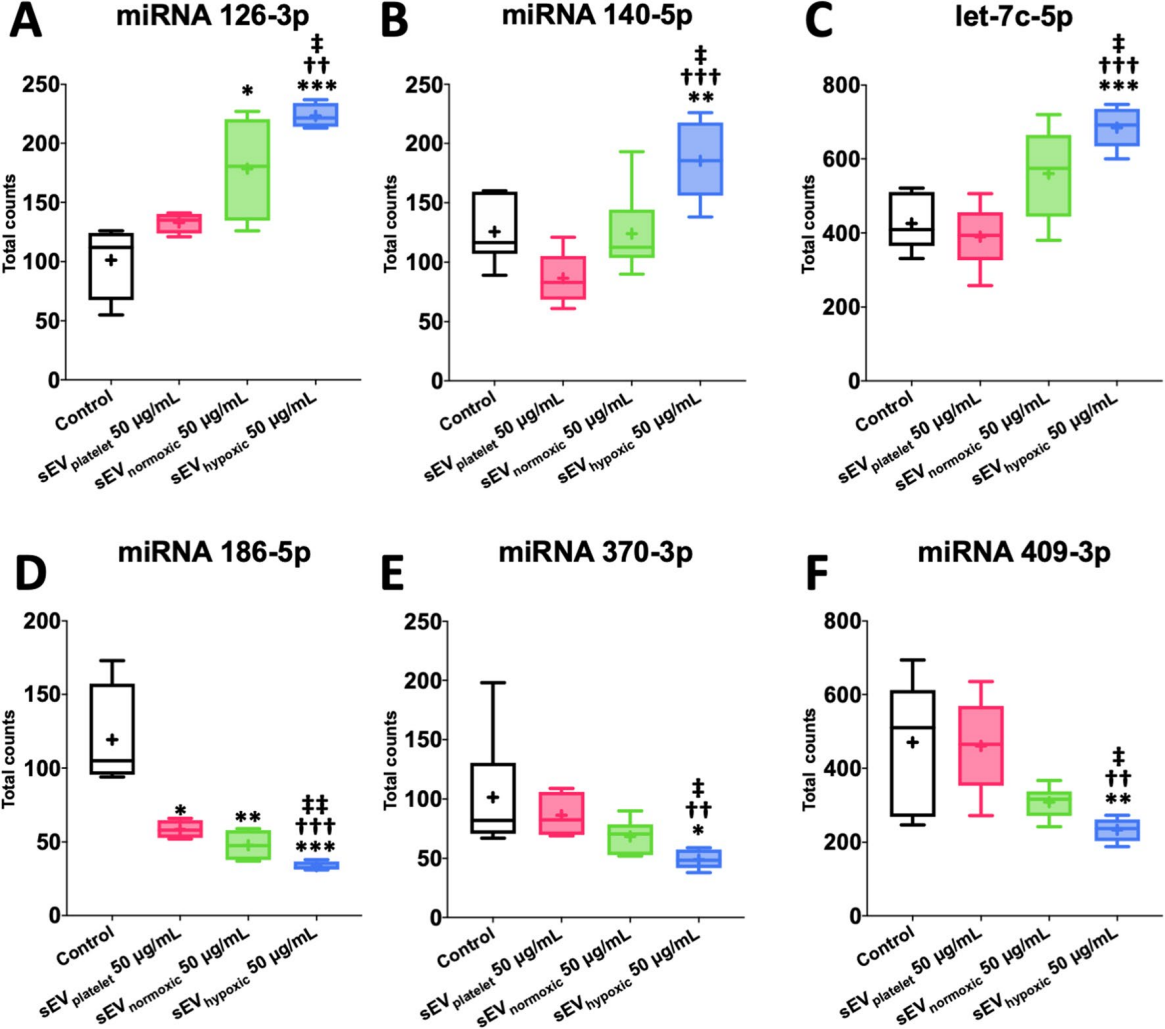
Cerebral microvascular endothelial cells exposed to sEVs obtained from hypoxic MSCs exhibit a distinct microRNA signature associated with angiogenesis. Total counts of **A** miR-126-3p, **B** miR-140-5p, **C** let-7c-5p, **D** miR-186-5p, **E** miR-370-3p and **F** miR-409-3p, evaluated by NanoString analysis, in hCMEC/D3 cells exposed to control conditions, sEVs obtained from MSC culture media that contain platelet lysates (50 µg/mL; sEV_platelet_), sEVs obtained from MSCs ( source 41.5) cultured under regular ‘normoxic’ conditions (21% O_2_; 50 µg/mL; sEV_normoxic_) or sEVs obtained from MSCs (source 41.5) cultured under hypoxic conditions (1% O_2_; 50 µg/mL; sEV_hypoxic_). Data are box plots with medians (lines inside boxes)/means (crosses inside boxes) ± interquartile ranges (IQR; boxes) with minimum/maximum values as whiskers (*n* = 4–6 samples per group). *p < 0.05, **p < 0.01, ***p < 0.001 compared with control/^††^*p* < 0.01, ^†††^*p* < 0.001 compared with sEV_platelet_/^‡^*p* < 0.05, ^‡‡^*p* < 0.01 compared with sEV_normoxic_

To identify mechanisms via which sEVs promote cerebral angiogenesis, we exposed hCMEC/D3 cells to control conditions (no sEV exposure), sEV_platelet_, sEV_normoxic_ or sEV_hypoxic_ at the dose that had strongest effects in the endothelial proliferation, migration, and tube formation assays (50 µg/mL) and examined the effects of sEVs on miRNA levels by NanoString gene expression analysis. Of 800 miRNAs examined, 6 miRNAs were differentially regulated by sEV_hypoxic_ compared with control conditions not receiving sEVs, sEV_platelet_ and sEV_normoxic_. Three of these were upregulated (miR-126-3p, miR-140-5p, let-7c-5p, Fig. 5A–C) and three were downregulated (miR-186-5p, miR-370-3p, miR-409-3p; Fig. 5D–F) by sEV_hypoxic_. KEGG pathway analysis by means of miRPathDB 2.0 (https://mpd.bioinf.uni-sb.de/mirnas.html; accessed April 20th, 2021) revealed that these miRNAs were associated with neurotrophin signaling (miR-126-3p, let-7c-5p), focal adhesion (miR-126-3p, miR-186-5p), VEGF signaling (miR-126-3p), leukocyte transendothelial migration (miR-126-3p), adherens junction (miR-409-3p) and cancer (miR-126-3p, miR-140-5p, let-7c-5p) pathways.

### sEV preparations obtained from hypoxic MSCs are enriched for growth factor pathway-associated proteins and extracellular matrix proteins/proteases and reduced for proteins involved in oxidative metabolism

To identify proteins in sEVs obtained from hypoxic MSCs that confer angiogenic properties, we compared the proteome of sEV_hypoxic_ and sEV_normoxic_ by LC/MS–MS. 52 proteins were differentially abundant in sEV_hypoxic_ and sEV_normoxic_ preparations. 19 were significantly enriched and 33 significantly reduced in sEV_hypoxic_. These proteins are summarized in Tables 1 and 2. Pathway analysis via KEGG Mapper (https://www.genome.jp/kegg/tool/map_pathway1.html; accessed April 21st, 2021) showed that proteins enriched in sEV_hypoxic_ compared with sEV_normoxic_ preparations were involved in (a) extracellular matrix (ECM)–receptor interaction (collagen alpha-1(VI) chain [COL6A1], collagen alpha-2(VI) chain [COL6A2], collagen alpha-3(VI) chain [COL6A3], tenascin-C [TNC]), (b) focal adhesion (COL6A1, COL6A2, COL6A3, TNC), (c) leukocyte transendothelial migration (72 kDa type IV collagenase [MMP2]), (d) protein digestion and absorption (collagen alpha-1(V) chain [COL5A1], COL6A1, COL6A2, COL6A3, collagen alpha-1(XII) chain [COL12A1]) and (e) cholesterol metabolism (apolipoprotein A-II [APOA2], apolipoprotein A-IV [APOA4], angiopoietin-related protein-4 [ANGPTL4]). A number of growth factor-associated proteins (IGF binding protein-3 [IGFBP3], transforming growth factor [TGF]-β-induced protein ig-h3 [TGFBI], latent-TGF-β-binding protein-2 [LTBP2], EGF-like repeat and discoidin I-like domain-containing protein-3 [EDIL3]) were also found. On the contrary, proteins reduced in sEV_hypoxic_ compared with sEV_normoxic_ preparations were associated with (a) metabolic pathways (phosphatidylinositol-glycan-specific phospholipase D [GPLD1], 6-phosphogluconate dehydrogenase [PGD], peroxiredoxin-6 [PRDX6]), ECM–receptor interaction (fibronectin [FN1], von Willebrand factor [VWF]), (b) endocytosis (F-actin-capping protein subunit alpha-1 [CAPZA1]), (c) focal adhesion (FN1, VWF), (d) regulation of the actin cytoskeleton (FN1, kininogen-1 [KNG1]), (e) the complement system (mannan-binding lectin serine protease-2 [MASP2], heparin cofactor-2 [SERPIND1], KNG1, complement C1q subcomponent subunit-C [C1QC], complement C1s subcomponent [C1S], VWF) and platelet activation (tyrosine protein kinase Lyn [LYN], VWF).

**Table 1:** Proteins significantly enriched in sEV preparations obtained from MSCs cultured under hypoxic conditions

Protein (Gene name)	Gene ID	Ratio
72 kDa type IV collagenase (*MMP2*)	4313	2.609
Collagen alpha-2(VI) chain (*COL6A2*)	1292	2.506
Collagen alpha-3(VI) chain (*COL6A3*)	1293	2.472
Collagen alpha-1(VI) chain (*COL6A1*)	1291	2.447
Insulin-like growth factor-binding protein-3 (*IGFBP3*)	3486	2.322
Angiopoietin-related protein-4 (*ANGPTL4*)	51,129	2.213
Apolipoprotein A-IV (*APOA4*)	337	2.177
Collagen alpha-1(XII) chain (*COL12A1*)	1303	2.059
Transforming growth factor-beta-induced protein ig-h3 (*TGFBI*)	7045	1.962
Collagen alpha-1(V) chain (*COL5A1*)	1289	1.930
Latent transforming growth factor beta-binding protein-2 (*LTBP2*)	4053	1.895
Apolipoprotein A-II (*APOA2*)	336	1.883
Cell migration-inducing and hyaluronan-binding protein (*CEMIP*)	57,214	1.873
Serum amyloid A-4 protein (*SAA4*)	6291	1.806
Tenascin-C (*TNC*)	3371	1.682
HtrA serine protease (*HTRA1*)	5654	1.650
Lactadherin (*MFGE8*)	4240	1.613
Protein-lysine-6-oxidase (*PL6O*)	348,959	1.593
Epidermal growth factor-like repeat and discoidin I-like domain-containing protein-3 (*EDIL3*)	10,085	1.515

The column in the center presents gene IDs. The right column exhibits ratios of protein abundances in sEVs obtained from 41.5 MSCs cultured under hypoxic conditions (1% O_2_; sEV_hypoxic_) and sEVs obtained from MSCs cultured under regular ‘normoxic’ conditions (21% O_2_; sEV_normoxic_) determined by LC/MS–MS

**Table 2:** Proteins significantly reduced in sEV preparations obtained from MSCs cultured under hypoxic conditions

Protein (Gene name)	Gene ID	Ratio
Inter-alpha-trypsin inhibitor heavy chain H4 (*ITIH4*)	3700	0.208
Heparin cofactor-2 (*SERPIND1*)	3053	0.280
Alpha-2-HS glycoprotein (*AHSG*)	197	0.301
Kininogen-1 (*KNG1*)	3827	0.409
Lysyl oxidase homolog-4 (*LOXL4*)	84,171	0.422
Retinol-binding protein-4 (*RBP4*)	5950	0.427
Proteoglycan-4 (*PRG4*)	10,216	0.443
Signal peptide, CUB and EGF-like domain-containing protein-3 (*SCUBE3*)	222,663	0.456
Cholesteryl ester transfer protein (*CETP*)	1071	0.482
Secreted phosphoprotein-2 (*SPP2*)	6694	0.495
von Willebrand factor (*VWF*)	7450	0.498
Immunoglobulin heavy variable 4–28 (*IGHV4-28*)	28,400	0.552
Inter-alpha-trypsin inhibitor heavy chain H3 (*ITIH3*)	3699	0.576
Coiled-coil domain-containing protein-73 (*CCDC73*)	493,860	0.595
Mannan-binding lectin serine protease-2 (*MASP2*)	10,747	0.598
Lymphocyte antigen-6 complex locus protein G6f (*LY6G6f*)	259,215	0.605
Fibulin-1 (*FBLN1*)	2192	0.606
Peroxiredoxin-6 (*PRDX6*)	9588	0.607
Phosphatidylinositol-glycan-specific phospholipase D (*GPLD1*)	2822	0.610
Complement C1s subcomponent (*C1S*)	716	0.614
F-actin-capping protein subunit alpha-1 (*CAPZA1*)	829	0.617
Tyrosine protein kinase Lyn (*LYN*)	4067	0.618
Galectin-1 (*LGALS1*)	3956	0.643
Complement C1q tumor necrosis factor-related protein-3 (*C1QTNF3*)	114,899	0.649
Complement C1q subcomponent subunit-C (*C1QC*)	714	0.651
Kallistatin (*SERPINA4*)	5267	0.657
Haptoglobin (*HP*)	3240	0.657
Fibronectin (*FN1*)	2335	0.659
Procollagen C-endopeptidase enhancer-2 (*PCOLCE2*)	26,577	0.662
IgGFc-binding protein (*FCGBP*)	8857	0.662
Endoplasmin (*HSP90B1*)	7184	0.664
Carboxypeptidase N subunit-2 (*CPN2*)	1370	0.667
6-phosphogluconate dehydrogenase, decarboxylating (*PGD*)	5226	0.667

The column in the center presents gene IDs. The right column exhibits ratios of protein abundances in sEVs obtained from MSCs cultured under hypoxic conditions (1% O_2_; sEV_hypoxic_) and sEVs obtained from 41.5 MSCs cultured under regular ‘normoxic’ conditions (21% O_2_; sEV_normoxic_) determined by LC/MS–MS

### sEV preparations obtained from hypoxic MSCs increase microvascular density and long-term neuronal survival, and promote neurological recovery after focal cerebral ischemia in mice

**Fig. 6:**
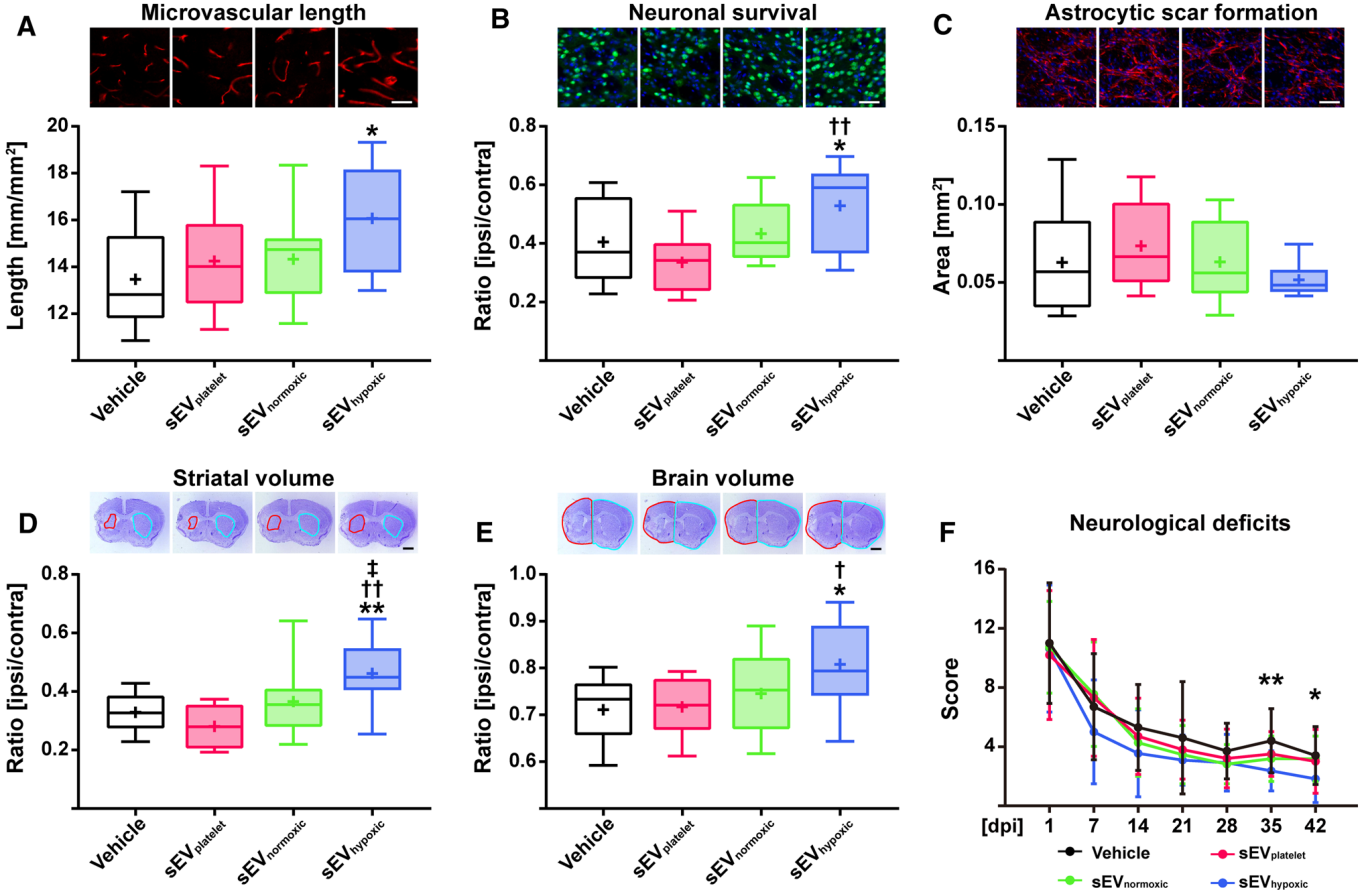
sEVs obtained from hypoxic MSCs induce post-ischemic angiogenesis, brain remodeling and neurological recovery in a mouse model of ischemic stroke. **A** Density of CD31^+^ cerebral microvessels in the previously ischemic striatum, **B** number of NeuN^+^ surviving neurons in the previously ischemic striatum and **C** area of GFAP^+^ astrocytic scar in the brain infarct at the rostrocaudal level of the bregma, which is the core of the middle cerebral artery territory, as well as **D** striatum volume, **E** whole-brain volume and **F** neurological deficits evaluated using the Clark score of mice exposed to 40 min middle cerebral artery occlusion (MCAO), which were intravenously treated after 24 h, 72 h and 120 h with vehicle (normal saline), sEVs obtained from MSC culture media that contain platelet lysate (sEV_platelet_), sEVs released by MSCs ( source 41.5) cultured under regular ‘normoxic’ conditions (21% O_2_; sEV_normoxic_; equivalent released by 2 × 10^6^ cells) or sEVs released by MSCs (source 41.5) cultured under hypoxic conditions (1% O_2_; sEV_hypoxic_; equivalent released by 2 × 10^6^ cells) followed by animal sacrifice after 56 days. Representative microphotographs are also shown. Data are box plots with medians (lines inside boxes)/means (crosses inside boxes) ± IQR (boxes) with minimum/maximum values as whiskers (in **A**–**E**) or mean ± SD values (in **F**) (*n* = 10 animals vehicle, 6 animals sEV_platelet_, 9 animals sEV_normoxic_, 9 animals sEV_hypoxic_). *p < 0.05, **p < 0.01 compared with control/^†^*p* < 0.05, ^††^*p* < 0.01 compared with sEV_platelet_/^‡^*p* < 0.05 compared with sEV_normoxic_. Scale bars: 50 µm (in **A**–**C**)/1 mm (in **D**, **E**)

To evaluate, if the angiogenic effect of sEVs obtained from hypoxic MSCs translates into enhanced post-stroke angiogenesis and brain remodeling in vivo, mice were exposed to transient intraluminal MCAO followed by delivery of vehicle, sEV_platelet_, sEV_normoxic_ (equivalent released by 2 × 10^6^ cells, in normal saline; MSC source 41.5, preparation A) or sEV_hypoxic_ (equivalent released by 2 × 10^6^ cells, in normal saline; MSC source 41.5, preparation A) at 24, 72 and 120 h post-ischemia. Neurological recovery was evaluated by Clark scores. After animal sacrifice at 56 days post-ischemia, microvascular length, neuronal survival and glial scar formation were immunohistochemically evaluated on coronal 20 µm sections in the ischemic striatum and cortex at the rostrocaudal level of the bregma, which represents the core of the vascular territory of the middle cerebral artery. Importantly, sEV_hypoxic_, but not sEV_platelet_ or sEV_normoxic_ increased microvascular length in the previously ischemic striatum (Fig. 6A), which is most vulnerable to brain injury in the intraluminal MCAO model, and the peri-infarct parietal cortex (Suppl. Fig. 9A), but not the infarct-remote motor cortex (Suppl. Fig. 9B). Moreover, sEV_hypoxic_, but not sEV_platelet_ or sEV_normoxic_ increased neuronal survival in the ischemic striatum (Fig. 6B), but did not influence astrocytic scar formation around the brain infarct (Fig. 6C), which were evaluated as surrogates of brain parenchymal remodeling. Of note, brain tissue protection by sEV_hypoxic_ was associated with an increased volume of the ischemic striatum (Fig. 6D) and ischemic brain hemisphere (Fig. 6E) at 56 days post-MCAO and enhanced neurological recovery (Fig. 6F). LDF recordings during and after MCAO did not differ between groups (Suppl. Fig. 9C). Our data demonstrate a robust neurovascular restorative signature induced by hypoxia-conditioned MSC-sEV preparations.

**Fig. 7:**
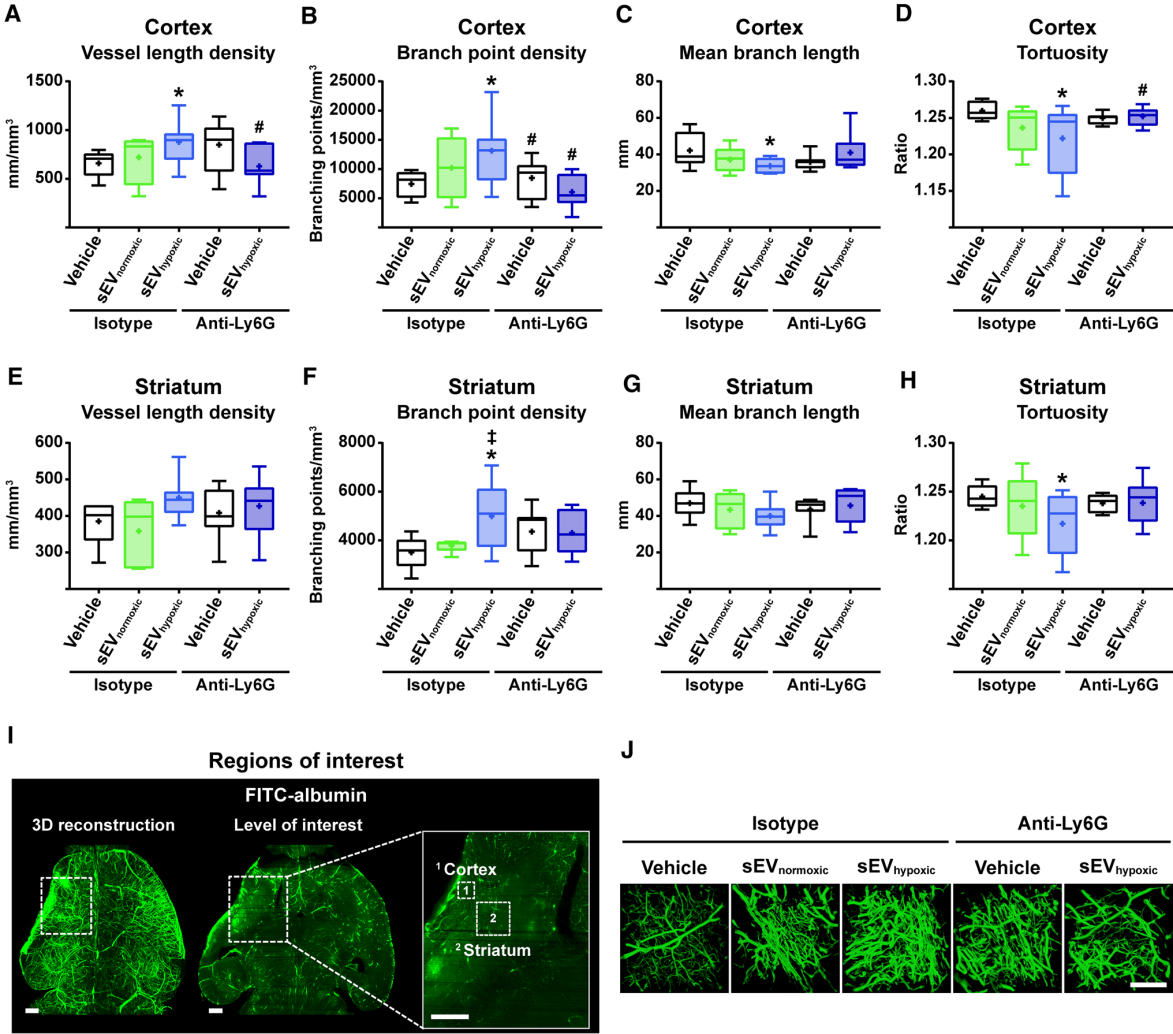
sEVs obtained from hypoxic MSCs increase microvascular remodeling following ischemic stroke, which is abolished in the absence of polymorphonuclear neutrophils (PMNs) in vivo. Microvascular network characteristics, that is, **A**, **E** microvascular length density, **B**,** F** branch point density, **C**,** G** mean branch length, and **D**,** H** tortuosity in the previously ischemic **A–D** cerebral cortex and **E–H** striatum evaluated by 3D light sheet microscopy in mice exposed to 40 min MCAO followed by 14 days survival, in which vehicle (normal saline), sEVs released by MSCs ( source 41.5) cultured under ‘normoxic’ conditions (21% O_2_; sEV_normoxic_; equivalent released by 2 × 10^6^ cells) or sEVs released by MSCs (source 41.5) cultured under hypoxic conditions (1% O_2_; sEV_hypoxic_; equivalent released by 2 × 10^6^ cells) were intravenously administered at 1, 3, and 5 days post-MCAO, while control (isotype) IgG or anti-Ly6G (clone 1A8; Ly6G indicates lymphocyte antigen-6, locus G) antibody was intraperitoneally applied at 1, 3, 5, and 7 days post-MCAO. **I** Representative axial overview images of a vehicle-treated mouse brain with magnifications depicting the regions of interest in the ischemic striatum and cortex, in which microvascular network characteristics were determined. **J** Representative maximum intensity projection (MIP) images in the ischemic cortex for the five experimental groups. Data are box plots with medians (lines inside boxes)/means (crosses inside boxes) ± IQR (boxes) with minimum/maximum values as whiskers (*n* = 6 animals isotype/vehicle, 5 animals isotype/sEV_normoxic_, 8 animals isotype/sEV_hypoxic_, 7 animals anti-Ly6G/vehicle, 7 animals anti-Ly6G/sEV_hypoxic_). *p < 0.05 compared with isotype/vehicle/^‡^*p* < 0.05 compared with isotype/sEV_normoxic_/^#^*p* < 0.05 compared with isotype/sEV_hypoxic_. Scale bars: 500 µm (in **I**)/100 µm (in **J**)

In view of the elevation of microvascular density by sEV_hypoxic_, but not sEV_normoxic_, we subsequently performed an in depth analysis of microvascular network characteristics in 3D by LSFM in mice exposed to transient intraluminal MCAO treated with vehicle, sEV_normoxic_ (MSC source 41.5; as above) or sEV_hypoxic_ (MSC source 41.5; as above) at 24, 72 and 120 h post-MCAO. Since PMNs have previously been shown by us to mediate acute neuroprotective effects of MSC-sEVs [30], we concomitantly depleted PMNs in two subgroups by anti-Ly6G antibody delivery. Animals were sacrificed at 14 days post-MCAO. For imaging of the reperfusion status at the onset of MSC-sEV treatment, mice exposed to intraluminal MCAO which did not receive any treatment and which were sacrificed at 24 h post-MCAO were also examined. In these mice, a pronounced reduction of microvascular length density by 58.1% and 42.0%, respectively, was noted in the previously ischemic cortex and striatum alongside an even larger reduction of branching point density by 74.9% and 51.8%, respectively, in the previously ischemic cortex and striatum (Suppl. Table 2). Microvascular mean branch length and tortuosity were unchanged (Suppl. Table 2). Notably, sEV_hypoxic_, but not sEV_normoxic_ increased microvascular length and branching point density and reduced mean branch length and tortuosity in the previously ischemic brain tissue at 14 days post-MCAO (Fig. 7A–J). LDF recordings during and after MCAO did not differ between groups (Suppl. Fig. 10). Interestingly, PMN depletion by anti-Ly6G antibody abolished the effects of sEV_hypoxic_ on microvascular network characteristics (Fig. 7A–J).

## Discussion

We herein show that sEVs obtained from supernatants of MSCs cultured under hypoxic conditions (1% O_2_; sEV_hypoxic_), but not sEVs from cell culture media (sEV_platelet_) or sEVs from supernatants of MSCs cultured under regular ‘normoxic’ conditions (21% O_2_; sEV_hypoxic_) dose-dependently promote the proliferation, migration and tube formation of human cerebral microvascular endothelial cells belonging to the hCMEC/D3 cell line. Effects were reproducible for independent sEV preparations and donor sources. None of the sEVs influenced the viability of hCMEC/D3 cells cultured under regular ‘normoxic’ conditions. Yet, sEV_hypoxic_, but not sEV_platelet_ or sEV_normoxic_ enhanced the survival of hCMEC/D3 cells exposed to OGD followed by reoxygenation/recultivation. Mechanistically, sEV_hypoxic_ were found to regulate a distinct set of miRNAs in hCMEC/D3 cells, as shown by NanoString gene expression analysis, which were upregulated (miR-126-3p, miR-140-5p, let-7c-5p) or downregulated (miR-186-5p, miR-370-3p, miR-409-3p) by sEV_hypoxic_. Proteome analysis revealed 52 proteins that were differentially abundant in sEV_hypoxic_ and sEV_normoxic_, 19 of which were enriched and 33 reduced in sEV_hypoxic_. In an in vivo model of ischemic stroke in mice, that is, intraluminal MCAO, sEV_hypoxic_, but not sEV_platelet_ or sEV_normoxic_ increased microvascular length and branching point density in the previously ischemic striatum and cortex over up to 56 days, increased long-term neuronal survival, reduced brain atrophy and enhanced neurological recovery. Hence, sEV_hypoxic_ induced a robust brain remodeling response. In vivo, the proangiogenic effect of MSC-sEVs depended on the presence of PMNs. In PMN-depleted mice, sEV_hypoxic_ did not promote microvascular network remodeling.

Accordingly to this study, the restorative effects of MSC-derived sEV preparations on the proliferation, migration and tube formation of cerebral microvascular endothelial cells in vitro and post-ischemic angiogenesis and brain remodeling in the post-acute stroke phase in vivo differ from their effects in the acute stroke phase, in which our group has previously shown in the same mouse intraluminal MCAO model that sEV_hypoxic_ and sEV_normoxic_ very similarly reduced infarct volume and neuronal injury [30]. In this earlier study, sEV_hypoxic_, but not sEV_normoxic_ reduced blood–brain barrier permeability evaluated by IgG extravasation [30], suggesting that microvascular actions of sEVs are modified by hypoxic preconditioning. The prevention of PMN brain entry played a crucial role in the acute neuroprotective effects of MSC-sEVs [30]. In mice exhibiting PMN depletion by anti-Ly6G antibody delivery, MSC-sEVs did not have any additional effect on infarct volume, neuronal survival or the brain infiltration of monocytes and lymphocytes [30]. PMNs exacerbate ischemic injury in the acute stroke phase [15, 24]. While this detrimental role of PMNs is meanwhile well established [13], the proangiogenic role of PMNs in the post-acute stroke phase is new. Our data suggest a dual role of PMNs in the ischemic brain. The mechanisms underlying PMN-associated angiogenesis will deserve further studies. Besides the release of proteases and reactive oxygen species by PMNs that facilitate ECM remodeling, alterations in the PMN differentiation in response to sEVs might play a role.

The effects of MSC-sEVs on microvessels apparently depend on tissues and pathophysiological states. In cancer tissues, for example, MSC-sEVs may promote or inhibit angiogenesis depending on the precise MSC source and tumor microenvironment [6, 18, 41]. In cell culture, sEVs obtained from normoxic MSCs increased the tube formation of HUVECs in one [26], but not another [1] study. In the study exhibiting induction of tube formation by sEV_normoxic_ [26], signaling pathways important in wound healing (Akt, extracellular-regulated kinase-1/2, and signal transducer and activator of transcription-3) were activated and growth factors (hepatocyte growth factor, insulin-like growth factor-1, nerve growth factor, stromal-derived growth factor-1) were elevated by sEVs. A third study on MSC-sEVs in HUVECs described that the angiogenic effects of sEVs closely depended on the presence of HIF-1α in parental MSCs [9]. MSCs overexpressing HIF-1α were found to exhibit an increased abundance of the Notch ligand Jagged-1 in sEVs [9]. Jagged-1-containing sEVs increased angiogenesis in Matrigel-based tube formation and plug assays [9]. These effects were blocked by prior incubation of sEVs with an anti-Jagged-1 antibody. The observation that sEV_hypoxic_ specifically induce angiogenesis in cerebral microvessels to the best of our knowledge is new. sEVs obtained from ‘normoxic’ adipose tissue-derived MSCs have previously been shown to promote the migration and tube formation of primary rat brain microvascular endothelial cells exposed to OGD [38].

By means of NanoString expression analysis, we identified a set of miRNAs, which were upregulated (miR-126-3p, miR-140-5p, let-7c-5p) or downregulated (miR-186-5p, miR-370-3p, miR-409-3p) in hCMEC/D3 cells by sEV_hypoxic_. It has previously been noted that MSC-derived sEV_hypoxic_ exhibit higher miR-126 levels than sEV_normoxic_ [20]. In HUVECs, the elevated miR-126 levels conferred angiogenic effects of sEV_hypoxic_ [20]. miR-126 formation was HIF-1α-dependent. Indeed, HIF-1α knockdown decreased miR-126 levels in MSCs and MSC-sEVs, thereby abolishing their angiogenic properties [20]. In rats, sEVs from miR-126 overexpressing MSCs promoted angiogenesis and neurogenesis and reduced lesion volume after spinal cord injury [16]. miR-126 overexpressing sEVs promoted the migration and tube formation of HUVECs [16]. miR-140-5p, on the other hand, was previously found to be decreased in the ischemic brain of rats exposed to MCAO and hypoxic HUVECs [27]. In HUVECs, miR-140-5p transfection decreased endothelial proliferation, migration and tube formation probably by mechanisms involving VEGF-A transcription, as indicated by luciferase reporter assay studies [27]. Let-7 miRNAs are hypoxia-responsive miRNAs induced in a HIF1α and argonaute-1 (AGO1)-dependent way in HUVECs which promote angiogenesis, as shown in let-7a and let-7e overexpression and antagomir-mediated downregulation studies using Matrigel-based tube formation and plug assays [5]. miR-186 and miR-370 have anti-angiogenic effects in retinoblastoma [34] and human dermal microvascular endothelial cells, HUVEC spheroids and mouse aortic rings [11], respectively. miR-409-3p inhibits proliferation, vasculogenic mimicry and lung metastasis of HT1080 fibrosarcoma cells [31]. A role of the latter miRNAs in the proliferation, migration and tube formation of cerebral microvascular endothelial cells has so far not been shown.

Proteome analysis by LC/MS–MS revealed 52 proteins that were differentially abundant in sEV preparations obtained from MSC-derived sEV_hypoxic_ and sEV_normoxic_, 19 of which were enriched and 33 reduced. Among those proteins enriched in sEV_hypoxic_ were proteins involved in ECM–receptor interaction (COL6A1, COL6A2, COL6A3, TNC), focal adhesion (COL6A1, COL6A2, COL6A3, TNC), leukocyte transendothelial migration (MMP2), protein digestion and absorption (COL5A1, COL6A1, COL6A2, COL6A3, COL12A1) and cholesterol metabolism (APOA2, APOA4, ANGPTL4). A number of growth factor-associated proteins (IGFBP3, TGFBI, LTBP2, EDIL3) were also found. Enrichment of proteins belonging to the platelet-derived growth factor [PDGF], EGF and FGF pathways has previously been observed in sEV_hypoxic_ compared with sEV_normoxic_ [2]. Their study protocol differed from the present study in that the hypoxia stimulus was combined with fetal bovine serum starvation, mimicking conditions of growth factor deprivation in peripheral artery disease [2]. In their study, enrichment of various proteins belonging to the nuclear factor (NF)-κB and cholesterol/lipid biosynthesis pathways were furthermore noted [2]. Among the proteins identified, 72 kDa type IV collagenase (i.e., gelatinase-A, matrix metalloproteinase [MMP]-2) was also found [2]. Accumulation of the serine protease HTRA1 in MSC-sEVs has to be best of our knowledge not been shown. Among those proteins reduced in sEV_hypoxic_ were proteins involved in metabolic pathways (GPLD1, PGD, PRDX6), ECM–receptor interaction (FN1, VWF), endocytosis (CAPZA1), focal adhesion (FN1, VWF), regulation of the actin cytoskeleton (FN1, KNG1), the complement system (MASP2, SERPIND1, KNG1, C1QC, C1S, VWF) and platelet activation (LYN, VWF). The presence of coagulation and complement factors in MSC-sEVs has already been shown [8, 23]. In rat models of spinal cord injury, MSC-sEVs reduced complement activation [40]. Regarding the detection of coagulation and complement factors as components on MSC-sEVs, possible by-products enriched in sEVs originating from the plasma carefully need to be considered. Such by-products might contribute to the mode of action of sEVs [33] and perhaps confer proangiogenic activities [32]. Whatever the mode of action or the active components of MSC-EV preparations may be, this study provides robust evidence that hypoxia-preconditioning should increase the efficacy of MSC-sEV preparations in ischemic stroke recovery.

## Supplementary Information

Below is the link to the electronic supplementary material.

Supplementary file1 (DOCX 2803 kb)

## Data Availability

Data supporting this study will be made available to qualified researchers upon reasonable request.
