# Supplementary material for: Small extracellular vesicles obtained from hypoxic mesenchymal stromal cells have unique characteristics that promote cerebral angiogenesis, brain remodeling and neurological recovery after focal cerebral ischemia in mice
Source: Basic Res Cardiol. 2021 Jun 8;116(1):40. doi: 10.1007/s00395-021-00881-9 (PMC8187185; doi:10.1007/s00395-021-00881-9)
Supplement: Supplementary file 1 — Supplementary file1 (DOCX 2803 kb) [file 395_2021_881_MOESM1_ESM.docx]

**Small extracellular vesicles obtained from hypoxic mesenchymal stromal cells have unique characteristics that promote cerebral angiogenesis, brain remodeling and neurological recovery after focal cerebral ischemia in mice**

Jonas Gregorius, MSc^1^*, Chen Wang, MD^1^*, Oumaima Stambouli, MSc^2^, Tanja Hussner, MSc^1^, Yachao Qi, MSc^1^, Tobias Tertel, MSc^2^, Verena Börger, PhD^2^, Ayan Mohamud Yusuf, PhD^1^, Nina Hagemann, PhD^1^, Dongpei Yin, MSc^1^, Robin Dittrich, MSc^2^, Yanis Mouloud, MSc^2^, Fabian D. Mairinger, PhD^3^, Fouzi El Magraoui, PhD^4^, Aurel Popa-Wagner, PhD^5^, Christoph Kleinschnitz, MD^1^, Thorsten R. Doeppner, MD^6^, Matthias Gunzer, PhD^4,7^, Helmut E. Meyer, PhD^4,8^, Bernd Giebel, PhD^2^, Dirk M. Hermann, MD^1^. *Equal contribution. ^1^Department of Neurology and Center for Translational Neuro- and Behavioral Sciences (C-TNBS), ^2^Institute of Transfusion Medicine and ^3^Institute of Pathology, University Hospital Essen, University of Duisburg-Essen, Germany; ^4^Leibniz Institute for Analytical Sciences (ISAS), Dortmund, Germany; ^5^Center of Experimental and Clinical Medicine, University of Medicine and Pharmacy, Craiova, Romania, ^6^Department of Neurology, University Medicine Göttingen, Göttingen, Germany; ^7^Institute for Experimental Immunology and Imaging, University Hospital Essen, University of Duisburg-Essen, Germany; ^8^Medical Proteom-Center Ruhr University Bochum, Germany.

Number of supplemental figures: 10

Number of supplemental tables: 2

**Correspondence:**

Prof. Dirk M. Hermann, MD, Department of Neurology, University Hospital Essen, University of Duisburg-Essen, Hufelandstraße 55, D-45122 Essen, Germany; phone: +49-201-723-2180, e-mail: dirk.hermann@uk-essen.de

Prof. Bernd Giebel, PhD, Institute for Transfusion Medicine, University Hospital Essen, University of Duisburg-Essen; Hufelandstraße 55, D-45122 Essen, Germany; Phone: +49-201-723-4204, e-mail: bernd.giebel@uk-essen.de

**Supplemental Figures:**

**
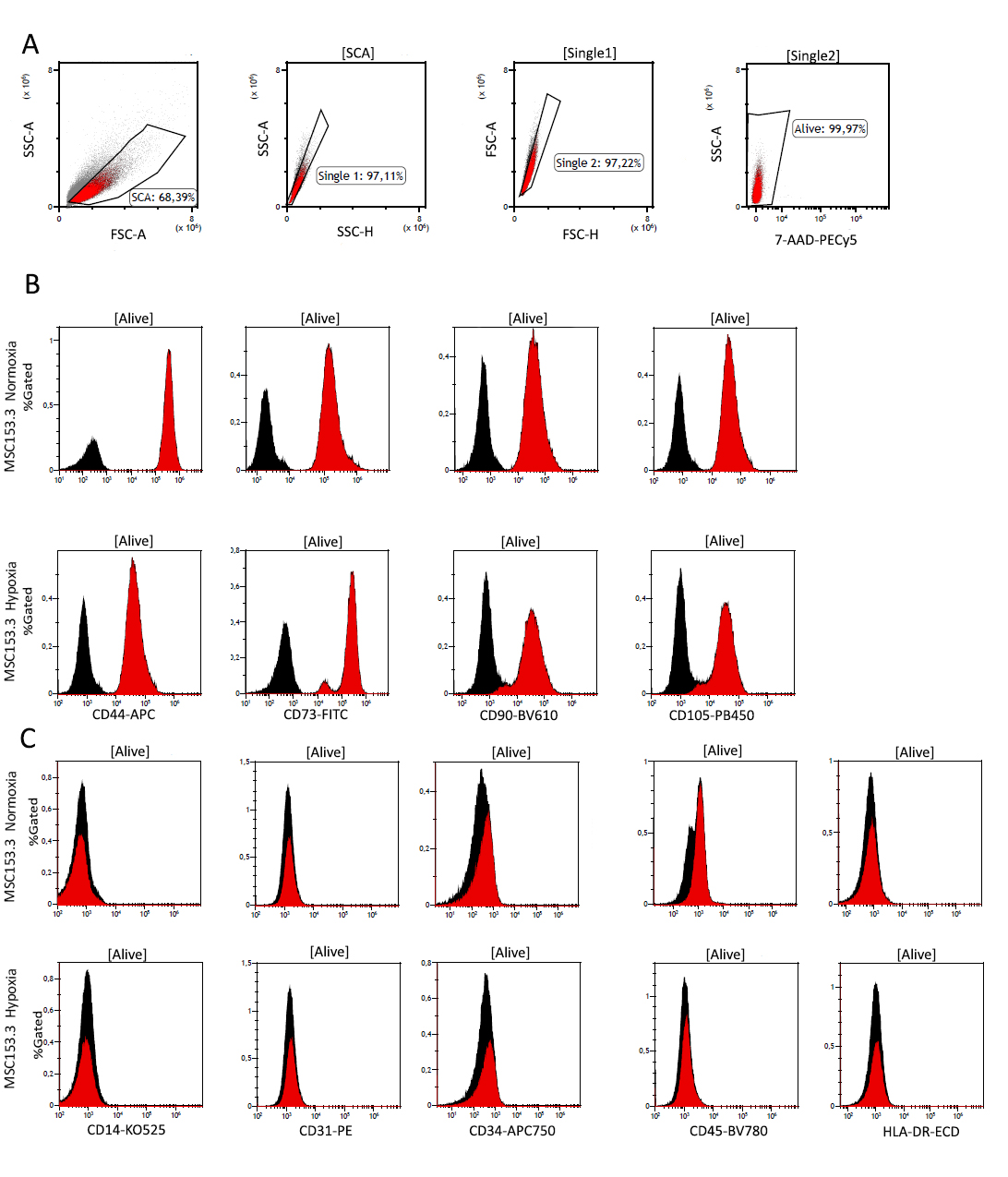
**

**Supplemental Figure 1:** **Mesenchymal stromal cells (MSCs) cultured under normoxic and hypoxic conditions express *bona fide* MSC markers.** (**A**) Gating strategy used for evaluating MSC markers. Scatters were gated and singlets were plotted by SSC-H and SSC-A and FSC-H and SSC-A. 7-AAD negative events were identified as live cells. Representative histograms of MSCs cultured under (**B**) ‘normoxic’ (21% O_2_) and (**C**) hypoxic (1% O_2_) conditions (MSC source 153.3). Black histograms represents unstained samples and red histograms represent stained samples.

**
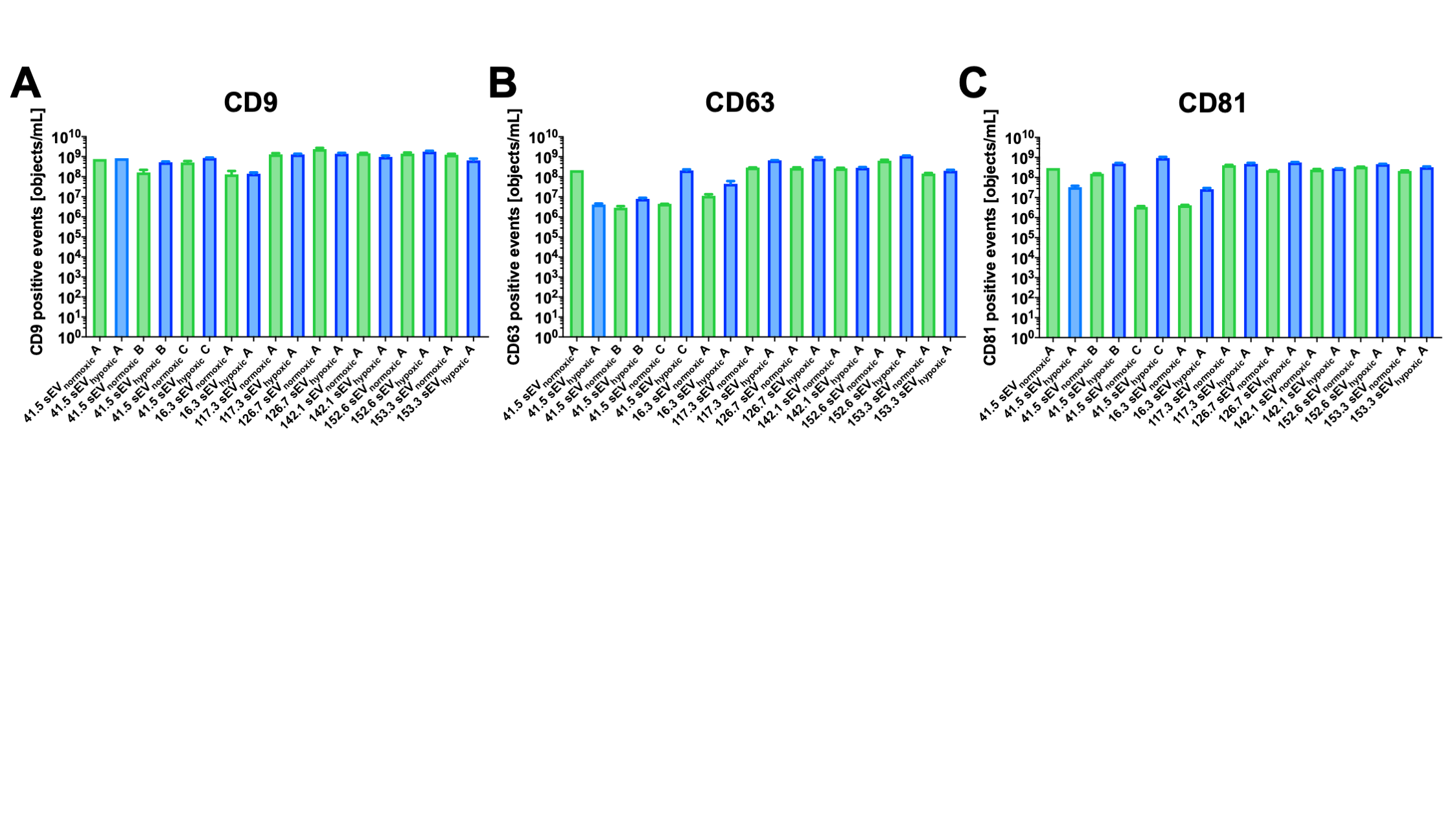
**

**Supplemental Figure 2: Flow cytometric characterization of MSC-derived small extracellular vesicle (sEV) preparations obtained under regular ‘normoxic’ and hypoxic conditions**. Number of (**A**) CD9^+^, (**B**) CD63^+^ and (**C**) CD81^+^ events in objects per milliliter tracked in different MSC-sEV preparations (labeled below x-axis as A, B and C) from various donors (sources 41.5, 16.3, 117.3, 126.7, 142.1, 152.6, and 153.3), which were cultured under regular ‘normoxic’ (21% O_2_; sEV_normoxic_) or hypoxic (1% O_2_; sEV_hypoxic_) conditions evaluated by AMNIS ImageStream. Data are mean ± SD values.

**
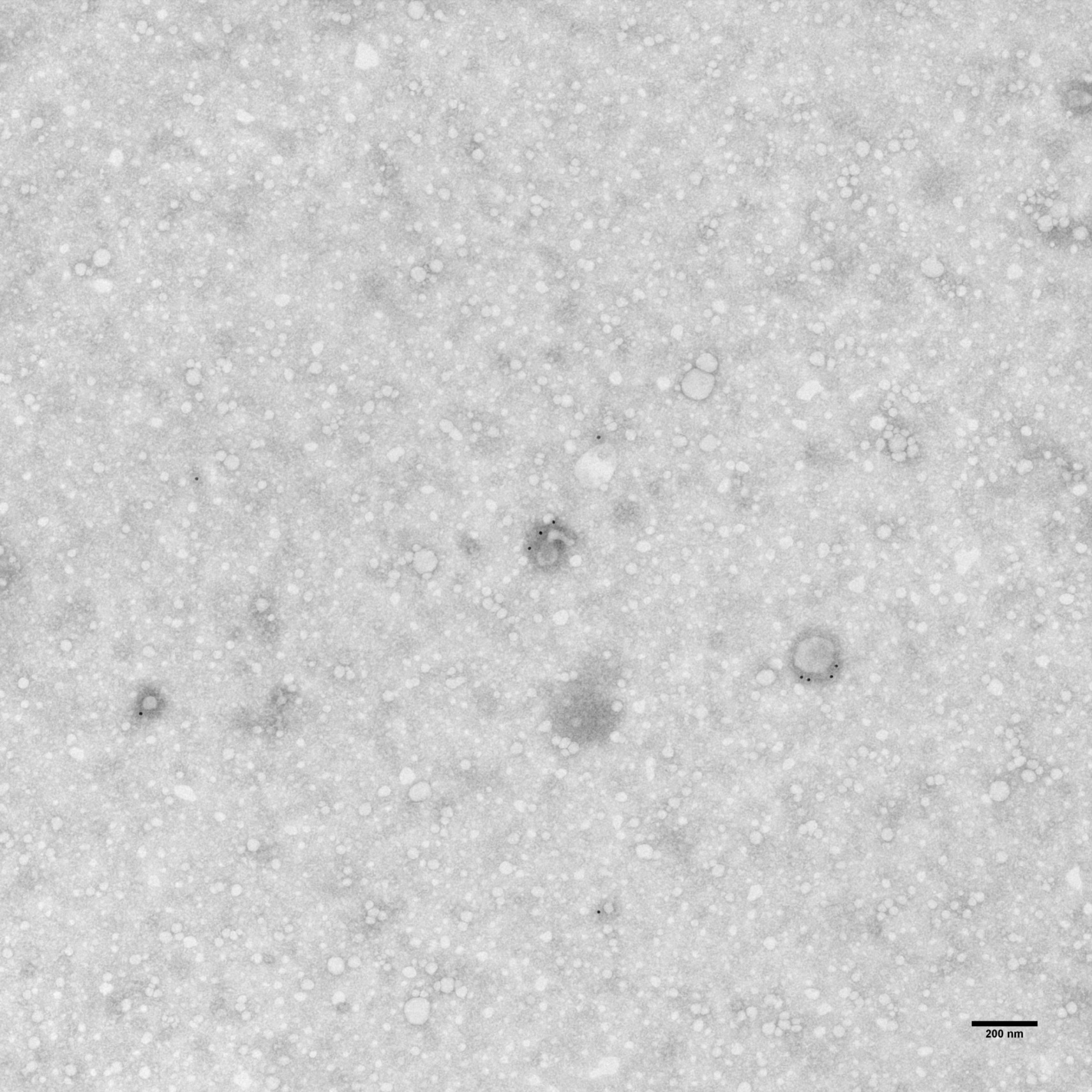
**

**Supplemental Figure 3: Transmission electron microscopy image of representative CD9^+^ small extracellular vesicle (sEVs) obtained from mesenchymal stromal cells (MSCs; source 41.5) cultured under ‘hypoxic’ conditions**. Sections were immunostained for CD9 (black dots). Scale bar, 200 nm.

**Supplemental Figure 4: sEVs obtained from 41.5 MSCs cultured under hypoxic conditions increase cerebral microvascular endothelial cell proliferation independent of the sEV preparation.** (**A-C**) Total number of proliferating human microvascular endothelial cells (hCMEC/D3) after exposure to control conditions or 50 µg/mL of each of three preparations (labeled preparations A, B and C) of sEVs obtained from MSC culture media that contain platelet lysates (sEV_platelet_), sEVs obtained from MSCs (source 41.5) cultured under regular ‘normoxic’ conditions (21% O_2_; sEV_normoxic_) or (**C**) sEVs obtained from MSCs (source 41.5) cultured under hypoxic conditions (1% O_2_; sEV_hypoxic_). Data are mean ± SD values (n=3 independent experiments [in (**A**)-(**C**)]). **p<0.01, ***p<0.001 compared with control; ^††^p<0.01, ^†††^p<0.001 compared with sEV_platelet_; ^‡‡^p<0.01, ^‡‡‡^p<0.001 compared with sEV_normoxic_.

**
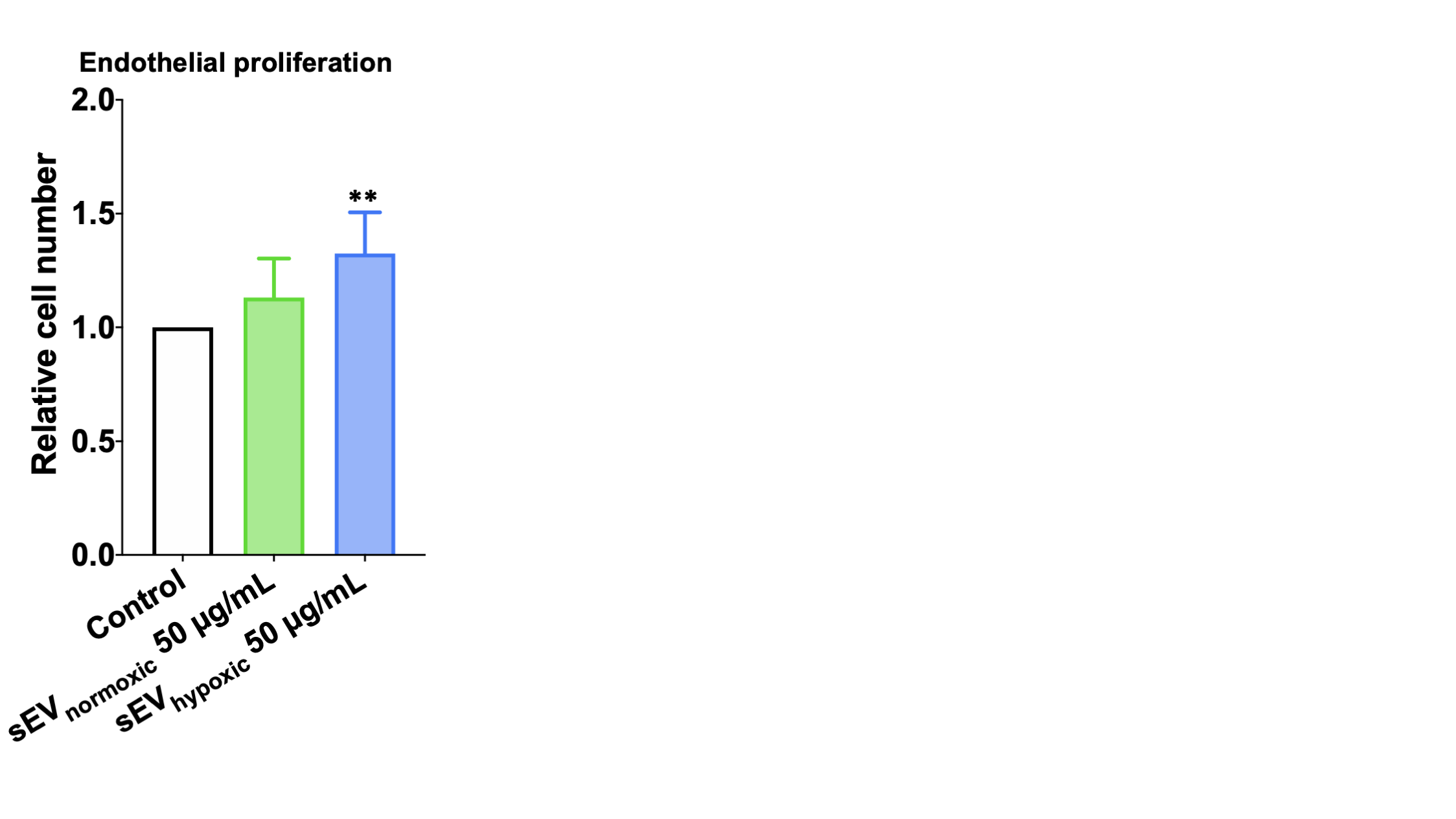
**

**Supplemental Figure 5: sEVs from 16.3 MSCs cultured under hypoxic conditions increase cerebral microvascular endothelial cell proliferation.** Relative number of proliferating hCMEC/D3 cells after exposure to control conditions, 50 µg/mL of sEVs obtained from MSCs (source 16.3) cultured under regular ‘normoxic’ conditions (21% O_2_; sEV_normoxic_) or 50 µg/mL of sEVs obtained from MSCs (source 16.3) cultured under hypoxic conditions (1% O_2_; sEV_hypoxic_). Data are mean ± SD values (n=4 independent experiments). **p<0.01 compared with control.

**Supplemental Figure 6: sEVs from hypoxic 41.5 MSCs increase cerebral microvascular endothelial cell migration independent of the sEV preparation.** (**A-C**) Total number of migrating hCMEC/D3 cells, determined in a transwell migration assay, after exposure to 50 µg/mL of each of three preparations (labeled preparations A, B and C) of sEVs obtained from MSC culture media that contain platelet lysates (sEV_platelet_), sEVs obtained from MSCs (source 41.5) cultured under ‘normoxic’ conditions (21% O_2_; sEV_normoxic_) or sEVs obtained from MSCs (source 41.5) cultured under hypoxic conditions (1% O_2_; sEV_hypoxic_). Data are mean ± SD values (n=3 independent experiments [in (**A**)-(**C**)]). *p<0.05, **p<0.01, ***p<0.001 compared with control; ^†^p<0.05, ^††^p<0.01, ^†††^p<0.001 compared with sEV_platelet_; ^‡^p<0.05, ^‡‡^p<0.01, ^‡‡‡^p<0.001 compared with sEV_normoxic_.

**Supplemental Figure 7: sEVs from hypoxic 41.5 MSCs increase cerebral microvascular endothelial tube formation independent of the sEV preparation.** (**A-C**) Total tube number, evaluated in a Matrigel-based tube formation assay, of hCMEC/D3 cells exposed to control conditions or 50 mg/mL of each of three preparations (labeled preparations A, B and C) of sEVs obtained from MSC culture media that contain platelet lysates (sEV_platelet_), sEVs obtained from MSCs (source 41.5) cultured under regular ‘normoxic’ conditions (21% O_2_; sEV_normoxic_) or sEVs obtained from MSCs (source 41.5) cultured under hypoxic conditions (1% O_2_; sEV_hypoxic_). Data are mean ± SD values (n=3 independent experiments [in (**A**)-(**C**)]). **p<0.01, ***p<0.001 compared with control/ ^††^p<0.01, ^†††^p<0.001 compared with sEV_platelet_/ ^‡‡^p<0.01, ^‡‡‡^p<0.001 compared with sEV_normoxic_.


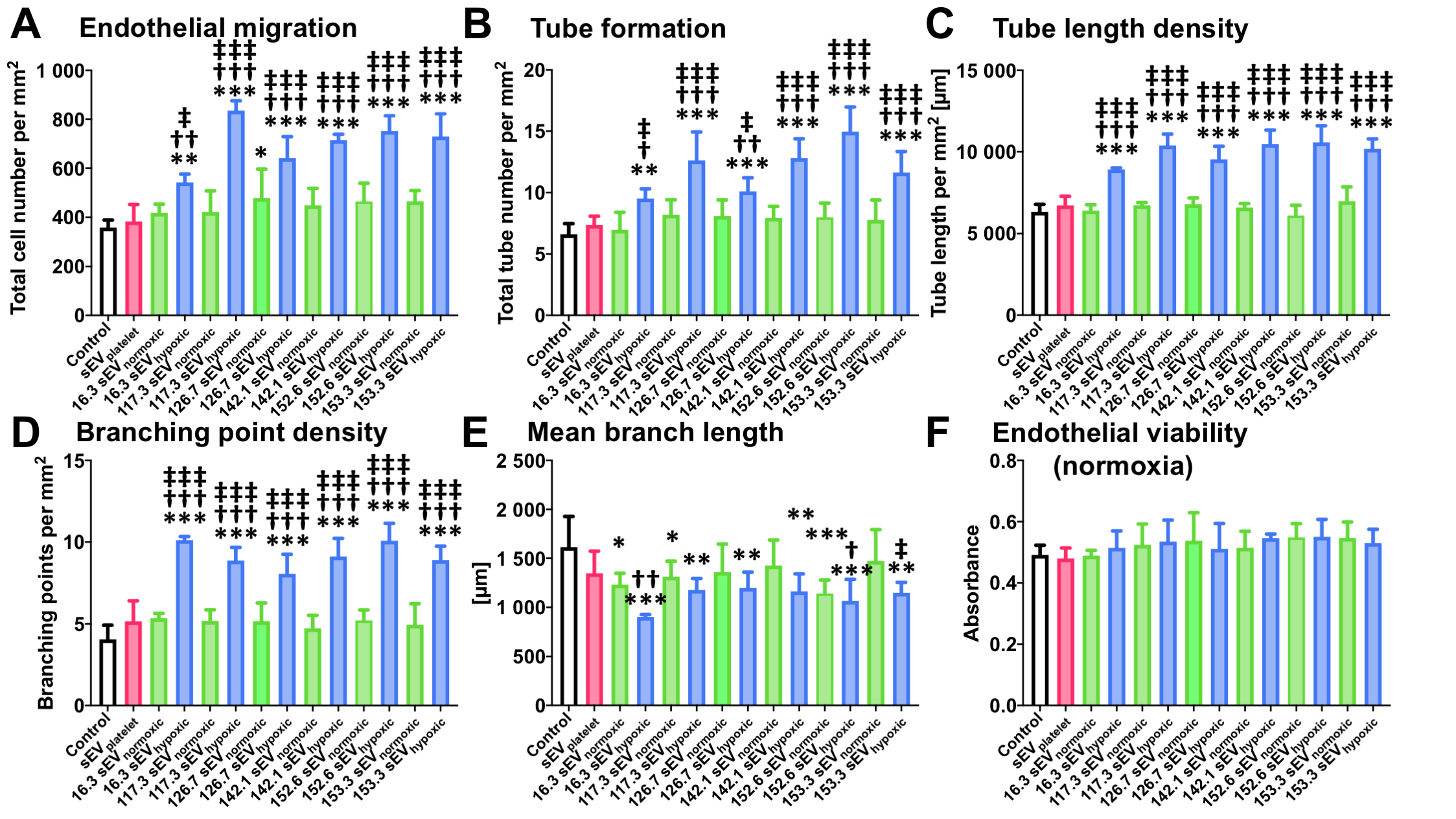


**Supplemental Figure 8: sEVs from hypoxic MSCs of different donors consistently increase the migration and tube formation of cerebral microvascular endothelial cells.** (**A**) Total number of migrating hCMEC/D3 cells, determined in a transwell migration assay, (**B**) tube number, (**C**) tube length density, (**D**) branching point density, and (**E**) mean branch length of hCMEC/D3 cells, evaluated in a Matrigel-based tube formation assay, as well as (**F**) endothelial viability, measured in a 3-(4,5-dimethylthiazol-2-yl)-2,5-diphenyltetrazolium bromide (MTT) assay in hCMEC/D3 cells cultured under regular ‘normoxic’ conditions (21% O_2_), which were exposed to control conditions, sEVs obtained from MSC culture media that contain platelet lysates (50 µg/mL; sEV_platelet_), sEVs obtained from various MSCs (sources 16.3, 117.3, 126.7, 142.1, 152.6 or 153.3) cultured under regular ‘normoxic’ conditions (21% O_2_; 50 µg/mL; sEV_normoxic_) or sEVs obtained from various MSCs (sources as above) cultured under hypoxic conditions (1% O_2_; 50 µg/mL; sEV_hypoxic_). Data are mean ± SD values (n=3 independent experiments [in (**A**)], 5 independent experiments [in (**B**)-(**E**)], 4 independent experiments [in (**F**)]). *p<0.05, **p<0.01, ***p<0.001 compared with control/ ^†^p<0.05, ^††^p<0.01, ^†††^p<0.001 compared with sEV_platelet_/ ^‡^p<0.05, ^‡‡^p<0.01, ^‡‡‡^p<0.001 compared with corresponding sEV_normoxic_.

**
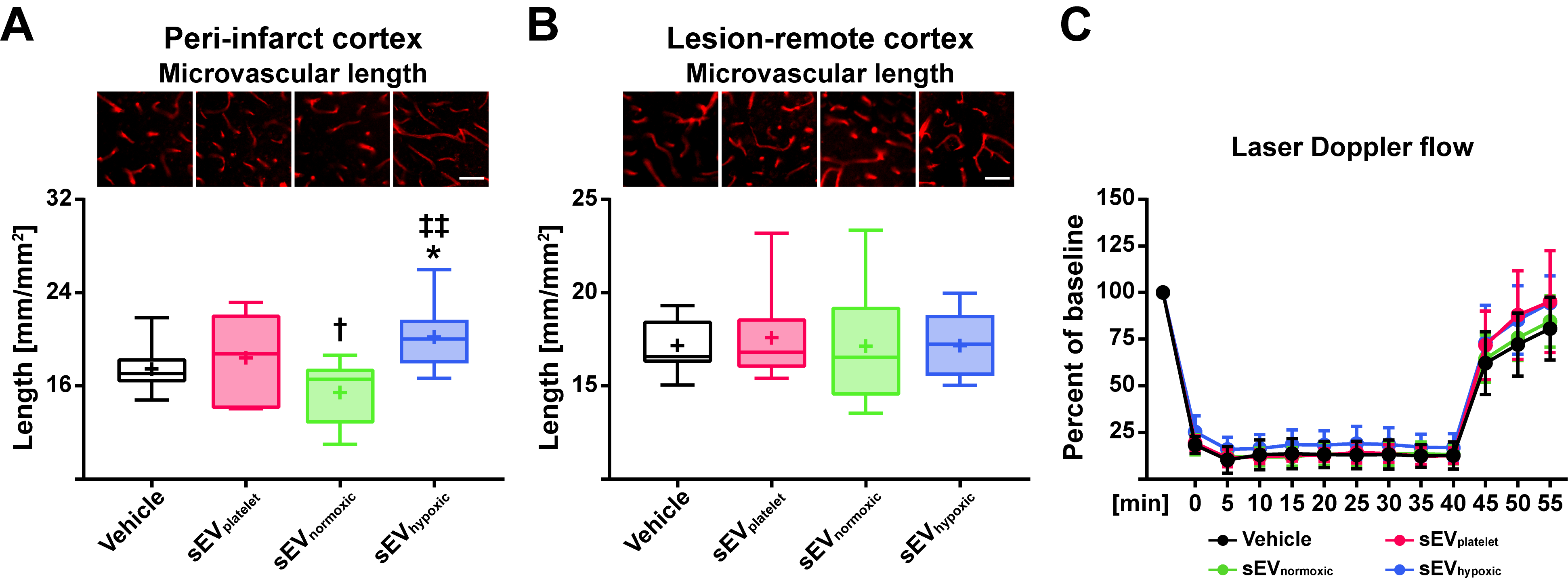
**

**Supplemental Figure 9: sEVs obtained from hypoxic 41.5 MSCs increase post-ischemic microvascular density in the peri-infarct cortex.** Density of CD31^+^ cerebral microvessels in the (**A**) peri-infarct parietal cortex and (**B**) infarct-remote motor cortex at the rostrocaudal level of the bregma, which is the core of the middle cerebral artery territory, of mice exposed to 40 min middle cerebral artery occlusion (MCAO), which were intravenously treated after 24 h, 72 h and 120 h with vehicle (normal saline), sEVs obtained from MSC culture media that contain platelet lysate (sEV_platelet_), sEVs released by MSCs (source 41.5) cultured under regular ‘normoxic’ conditions (21% O_2_; sEV_normoxic_; equivalent released by 2x10^6^ cells) or sEVs released by MSCs (source 41.5) cultured under hypoxic conditions (1% O_2_; sEV_hypoxic_; equivalent released by 2x10^6^ cells) followed by animal sacrifice after 56 days. Representative microphotographs are also shown. (**C**) Laser Doppler flow recording above the core of the middle cerebral artery territory during and after MCAO in the same groups. Data are box plots with medians (lines inside boxes)/ means (crosses inside boxes) ± interquartile ranges (boxes) with minimum/ maximum values as whiskers (in (**A**) and (**B**)) or mean ± SD values (in (**C**)) (n=10 animals vehicle, 6 animals sEV_platelet_, 9 animals sEV_normoxic_, 9 animals sEV_hypoxic_). *p<0.05 compared with control/ ^†^p<0.05 compared with sEV_platelet_/ ^‡‡^p<0.01 compared with sEV_normoxic_. Scale bar: 50 µm (in (**A**), (**B**)).

**
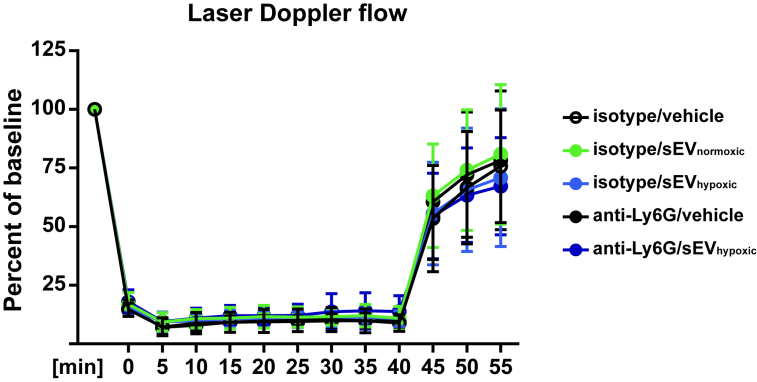
**

**Supplemental Figure 10: Transient ischemia followed by reproducible reperfusion in mice exposed to MCAO evaluated by 3D light sheet microscopy.** Laser Doppler flow recording above the core of the middle cerebral artery territory during and after 40 min MCAO of mice which were intravenously treated with vehicle (normal saline), sEVs released by MSCs (source 41.5) cultured under regular ‘normoxic’ conditions (21% O_2_; sEV_normoxic_; equivalent released by 2x10^6^ cells) or sEVs released by MSCs (source 41.5) cultured under hypoxic conditions (1% O_2_; sEV_hypoxic_; equivalent released by 2x10^6^ cells) at 1, 3, and 5 days post-MCAO, while control (isotype) IgG or anti-Ly6G (clone 1A8; Ly6G indicates lymphocyte antigen-6, locus G) antibody were intraperitoneally applied at 1, 3, 5, and 7 days post-MCAO. Data are mean ± SD values (n=6 animals isotype/vehicle, 5 animals isotype/sEV_normoxic_, 8 animals isotype/sEV_hypoxic_, 7 animals anti-Ly6G/vehicle, 7 animals anti-Ly6G/sEV_hypoxic_). No significant group differences were found.

**Supplemental Tables:**

**Supplemental Table 1: Characterization of MSC-sEV preparations by nanoparticle tracking analysis (NTA) and bicinchoninic acid assay (BCA).**

| MSC-sEV preparation | Particle concentration [particles/ ml] | Particle size [nm] | Protein concentration [µg/ µl] | Purity [particles/ mg protein] |
| --- | --- | --- | --- | --- |
| sEV_platelet_ (A) | 6.4x10^10^ | 137.1 | 7.29 | 4.7x10^10^ |
| sEV_platelet_ (B) | 3.3x10^11^ | 115.6 | 5.52 | 1.7x10^11^ |
| sEV_platelet_ (C) | 3.5x10^11^ | 107.7 | 9.59 | 3.7x10^10^ |
| 41.5 sEV_normox_ (A) | 1.48x10^11^ | 119.2 | 5.48 | 2.7x10^10^ |
| 41.5 sEV_normox_ (B) | 3.8x10^11^ | 125.6 | 7.84 | 4.8x10^10^ |
| 41.5 sEV_normox_ (C) | 1.6x10^11^ | 125.6 | 4.80 | 3.3x10^10^ |
| 41.5 sEV_hypox_ (A) | 8.40x10^10^ | 132.6 | 1.39 | 6.0x10^10^ |
| 41.5 sEV_hypox_ (B) | 2.80x10^11^ | 119.7 | 2.41 | 1.2x10^11^ |
| 41.5 sEV_hypox_ (C) | 1.36x10^11^ | 123.9 | 6.02 | 2.3x10^10^ |
| 16.3 sEV_normox_ (A) | 7.2x10^10^ | 120.9 | 0.62 | 1.2x10^11^ |
| 16.3 sEV_hypox_ (A) | 5.1x10^10^ | 110.4 | 0.89 | 5.7x10^10^ |
| 117.3 sEV_normox_ (A) | 2.0x10^11^ | 117.7 | 7.49 | 2.6x10^10^ |
| 117.3 sEV_hypox_ (A) | 4.0x10^11^ | 117.3 | 7.96 | 4.9x10^10^ |
| 126.7 sEV_normox_ (A) | 3.9x10^11^ | 118.4 | 10.44 | 1.8x10^10^ |
| 126.7 sEV_hypox_ (A) | 8.9x10^11^ | 112.0 | 16.16 | 5.5x10^10^ |
| 142.1 sEV_normox_ (A) | 3.3x10^11^ | 115.8 | 6.63 | 4.9x10^10^ |
| 142.1 sEV_hypox_ (A) | 4.7x10^11^ | 123.7 | 9.10 | 5.1x10^10^ |
| 152.6 sEV_normox_ (A) | 6.5x10^11^ | 135.0 | 6.76 | 9.6x10^10^ |
| 152.6 sEV_hypox_ (A) | 1.3x10^12^ | 137.6 | 18.81 | 6.9x10^10^ |
| 153.3 sEV_normox_ (A) | 2.3x10^11^ | 111.6 | 6.40 | 3.6x10^10^ |
| 153.3 sEV_hypox_ (A) | 2.1x10^11^ | 112.5 | 6.30 | 3.3 x10^10^ |

In the left column, letters (A) – (C) refer to independent MSC-sEV preparations.

**Supplemental Table 2. Microvascular network characteristics in the previously ischemic and contralateral non-ischemic cortex and striatum evaluated by 3D light sheet microscopy at 24 hours post-MCAO.**

|  | Vessel length density | | Branch point density | | Mean branch length | | Tortuosity | |
| --- | --- | --- | --- | --- | --- | --- | --- | --- |
|  | Cortex | Striatum | Cortex | Striatum | Cortex | Striatum | Cortex | Striatum |
| Ischemic | 324.1±  130.8* | 355.3±  134.4* | 2135.3±  957.7* | 2749.6±  1376.9** | 53.2±  14.4 | 45.6±  10.3 | 1.21±  0.06 | 1.21±  0.06 |
| Non-ischemic | 773.8±  240.6 | 612.6±  207.5 | 8497.6±  5354.8 | 5709.5±  2627.2 | 53.3±  13.0 | 48.2±  9.3 | 1.27±  0.02 | 1.25±  0.02 |

Data are means ± S.D. values (n=5 animals). *p<0.05, **p<0.01 compared with corresponding non-ischemic.
